# Academic self-concept and ecological predictors of longitudinal change in reading achievement in Chile: evidence from a national study

**DOI:** 10.3389/fpsyg.2026.1933540

**Published:** 2026-10-12

**Authors:** Sonia Salvo-Garrido, Ana Moraga-Pumarino, Manuel Mieres-Chacaltana, Karina Polanco-Levicán, Susan Avila Misciagno

**Affiliations:** 1 Departamento de Matemática y Estadística, Universidad de La Frontera, Temuco, Chile; 2 Departamento de Administración y Economía, Universidad de La Frontera, Temuco, Chile; 3 Departamento de Diversidad y Educación Intercultural, Facultad de Educación, Universidad Católica de Temuco, Chile; 4 Escuela de Psicología, Facultad de Ciencias Sociales y Educación, Universidad Santo Tomás, Temuco, Chile; 5 Department of Pharmaceutical and Clinical Sciences, Campbell University, Buies Creek, NC, United States

**Keywords:** academic self-concept, family reading attitudes, Indigenous status, reading achievement, reading trajectories, school climate and school violence, sex, socioeconomic status

## Abstract

**Background:**

Reading achievement is a foundational educational outcome, yet longitudinal evidence remains limited on how students’ reading achievement changes from primary to lower secondary education and how these changes are shaped by individual, family, school, and sociodemographic factors in unequal educational systems.

**Methods:**

This study examined longitudinal change in reading achievement from Grade 4 in 2015 to Grade 8 in 2019 in a national Chilean cohort of 123,827 students. Using linear mixed-effects models with repeated measures, we analyzed the contribution of students’ academic self-concept, academic engagement and self-efficacy, family reading attitudes, parental education, cultural capital, parental educational expectations, school climate, school violence, students’ self-reported sex, Indigenous status, and socioeconomic status.

**Results:**

Observed reading scores were lower at the Grade 8 assessment occasion than at the Grade 4 assessment occasion, with mean scores of 247.3 and 273.0 points, respectively, and the final model estimated a negative coefficient for the Grade 8 assessment indicator (*B* = −23.68, *p* < 0.001). Academic self-concept showed the largest positive association with reading achievement (*B* = 23.45, *p* < 0.001), followed by teacher regulation and normative guidance, parental educational expectations, parental education, classroom order and engagement, and family reading attitudes. School violence and peer aggression, perceived discrimination at school, and student–teacher aggression were negatively associated with achievement. The most parsimonious moderation model indicated that longitudinal change differed by the joint combination of time, sex, and Indigenous status, whereas socioeconomic status was mainly associated with achievement levels rather than change over time.

**Conclusion:**

These findings support an ecological and equity-oriented understanding of reading development, showing that longitudinal change in reading achievement is associated with academic self-beliefs, family literacy environments, school experiences, and structural inequalities.

## 1 Introduction

### 1.1 Longitudinal change in Reading achievement

Reading achievement is a foundational educational outcome because it enables students to understand, interpret, evaluate, and critically engage with written texts across school subjects and everyday contexts. Reading is understood as a complex process through which students construct meaning by integrating decoding and word recognition with comprehension, inference generation, and coherence formation (Ohle-Peters et al., 2023; Preusler et al., 2022). It also supports learning in mathematics, science, and social studies by enabling access to disciplinary knowledge and participation in school learning (Capin et al., 2022; Claes et al., 2024; Hay and Stevenson, 2024).

Reading achievement is closely linked to students’ educational trajectories and later opportunities, functioning as a cumulative educational resource that supports increasingly complex learning experiences across schooling (Levlin et al., 2024; Vasalampi et al., 2023). Differences acquired during primary education may therefore shape broader academic development. Higher reading proficiency has been associated with academic progression, educational track placement, attainment, and sustained engagement, whereas persistent reading difficulties may restrict later learning opportunities and contribute to lower academic self-confidence, motivational decline, and school disengagement (Cubillos and Troncoso, 2025; Larsen et al., 2022; Levlin et al., 2024; Torppa et al., 2024; Vasalampi et al., 2023). This is especially relevant in educational systems marked by social, territorial, and cultural inequalities (Claes et al., 2024; Cubillos and Troncoso, 2025; Yu et al., 2025).

Reading development should be understood as dynamic and cumulative rather than static. Longitudinal studies show that students do not follow uniform growth patterns; trajectories may reflect early advantages, accumulated disadvantages, and changing motivational, family, instructional, and contextual conditions (Larsen et al., 2022; Maclean et al., 2024; Shen and Goodrich, 2024; Shi et al., 2023). This aligns with cumulative advantage and disadvantage, or the Matthew effect, whereby early literacy gaps may persist or widen without timely academic and contextual support (Larsen et al., 2022; Levlin et al., 2024; Shen and Goodrich, 2024).

The transition from primary to lower secondary education is especially important because students encounter more complex texts, greater academic demands, specialized instruction, and changing relationships with teachers, peers, and schools (Larsen et al., 2022; Levlin et al., 2024; Preusler et al., 2022). Reading growth may also decelerate toward adolescence as instruction shifts from decoding to comprehension, critical interpretation, and disciplinary reading (Larsen et al., 2022; Ohle-Peters et al., 2023; Preusler et al., 2022; Shen and Goodrich, 2024).

Chilean evidence reinforces this perspective. Using SIMCE data from Grade 4, Grade 8, and Grade 10, Cubillos and Troncoso (2025) found temporal stability in reading achievement and motivation, while showing that their direction of influence may change across schooling: achievement predicted later motivation from Grade 4 to Grade 8, whereas motivation became more relevant for later achievement during secondary education. This is consistent with evidence that reading achievement, motivation, self-beliefs, and engagement are dynamically related over time (Cubillos and Troncoso, 2025; Symes et al., 2023; Torppa et al., 2024; Vasalampi et al., 2023).

From an ecological perspective, reading development is embedded in multiple systems of influence. Student characteristics, home literacy practices, parental expectations, cultural capital, school climate, teacher support, peer relations, and socioeconomic conditions shape the educational ecology in which reading development occurs (Bronfenbrenner, 1979; Chen et al., 2021; Claes et al., 2024; Mudrák et al., 2020; Yu et al., 2025). This view is particularly relevant in inequitable contexts, where opportunities to develop reading skills may differ by socioeconomic status, family resources, school composition, instructional quality, and adverse school experiences (Baysu et al., 2023; Bravo-Sanzana et al., 2022; Gubbins and Otero, 2020; Salvo-Garrido et al., 2023; Xiao et al., 2024).

Despite growing interest in educational trajectories, much evidence remains cross-sectional or focused on isolated predictors. Cross-sectional designs provide snapshots of performance and cannot distinguish stable between-student differences from within-student change (Claes et al., 2024; Larsen et al., 2022; Maclean et al., 2024; Tang et al., 2022). Longitudinal studies integrating individual, family, school, and sociodemographic factors are also scarce in Latin America. Examining longitudinal change in reading achievement in Chile using large-scale longitudinal data can therefore clarify how inequalities emerge, persist, or change between primary and lower secondary education, while identifying the relative contribution of psychological, family, school, and structural factors (Bravo-Sanzana et al., 2022; Cubillos and Troncoso, 2025; Gubbins and Otero, 2020; Salvo-Garrido et al., 2023).

### 1.2 Academic self-concept, motivation, and reading achievement

Among the psychological factors associated with reading development, academic self-beliefs and motivation have received considerable attention. Academic self-concept refers to students’ perceptions of their academic competence, whereas self-efficacy refers to beliefs about their capacity to perform specific academic tasks (Bandura, 1986; Basarkod et al., 2023; Geng et al., 2023). In reading, self-concept includes perceived competence, confidence, and perceived task difficulty, and has been identified as a central correlate of reading achievement, engagement, and persistence (Basarkod et al., 2023; Ramazan et al., 2023; Vasalampi et al., 2023; Wang, 2023).

Reading self-concept is multidimensional and domain-specific, with perceived competence and perceived difficulty varying by proficiency level (Basarkod et al., 2023). This aligns with expectancy-value and social cognitive perspectives, which emphasize that expectations of success, perceived competence, interest, and task value shape academic behavior and achievement (Bandura, 1986; Basarkod et al., 2023; Geng et al., 2023; Wang, 2023). Competent self-perceptions may promote engagement with texts, persistence, self-regulation, and sustained effort, contributing to both immediate performance and long-term reading trajectories (Geng et al., 2023; Vasalampi et al., 2023; Wang, 2023).

The relationship between academic self-concept and achievement is generally reciprocal: achievement experiences may strengthen perceived competence, while positive self-perceptions may promote effort, persistence, engagement, and subsequent achievement (Basarkod et al., 2023; Cubillos and Troncoso, 2025; Symes et al., 2023; Wang, 2023). Chilean longitudinal evidence also shows that reading achievement and motivation influence one another over time, although their direction and strength vary across schooling (Cubillos and Troncoso, 2025). Thus, academic self-concept should be interpreted within a developmental process in which prior achievement, motivation, instructional experiences, and contextual conditions mutually reinforce one another (Cubillos and Troncoso, 2025; Symes et al., 2023; Torppa et al., 2024; Vasalampi et al., 2023).

Self-beliefs may also explain how contextual factors are translated into academic outcomes. Ma et al. (2023) found that reading self-concept mediated the association between socioeconomic status and reading achievement in China and the United States, while teacher support moderated parts of this relationship. Ma et al. (2022) also showed that teacher feedback was associated with reading achievement through reading self-concept, and that disciplinary climate contributed to reading self-perceptions. These findings align with evidence that reading self-efficacy, confidence, teacher support, and school climate are components of systemic models of reading literacy (Chen et al., 2021; Ma et al., 2022; Ma et al., 2023; Mudrák et al., 2020; Xiao et al., 2024), suggesting that academic self-concept may link contextual advantages and disadvantages to reading achievement differences.

Academic self-concept is closely related to reading engagement, including behavioral, cognitive, and affective involvement in reading activities such as time spent reading, persistence, strategy use, interest, and enjoyment (Wang, 2023; Wang and Hu, 2025). Adolescents with higher reading self-concept tend to display stronger engagement profiles, which are associated with higher reading literacy (Vasalampi et al., 2023; Wang, 2023; Wang and Hu, 2025). This is particularly relevant during the transition to lower secondary education, when students face more demanding texts and may experience declines in motivation, perceived competence, and school engagement (Raufelder et al., 2024; Symes et al., 2023; Torppa et al., 2024; Wang, 2023).

These variables are also relevant for understanding reading differences by student-reported sex. Girls tend to outperform boys in reading and often report more positive reading attitudes, stronger reading-related motivation, greater enjoyment, and more frequent use of reading strategies (Acar-Erdol and Akin-Arikan, 2022; Huang and Chen, 2019; Reinhold et al., 2026; Taraszow et al., 2024). These differences should not be interpreted as fixed ability differences, but as developmental patterns shaped by motivation, self-beliefs, socialization, engagement, and domain-specific academic identities (Acar-Erdol and Akin-Arikan, 2022; Reinhold et al., 2026; Steinmann and Strietholt, 2026).

Chilean evidence further supports the importance of self-beliefs and motivation. Using SIMCE data, Salvo-Garrido et al. (2023) found that self-efficacy was one of the most relevant factors explaining variability in Mathematics and Language scores, followed by parental expectations and school-related variables. Together with international evidence, these findings suggest that longitudinal change in reading achievement may be associated with prior achievement, family resources, school conditions, perceived academic competence, and students’ motivational relationship with reading and school learning (Chen et al., 2021; Cubillos and Troncoso, 2025; Mudrák et al., 2020; Salvo-Garrido et al., 2023).

Taken together, this evidence supports considering academic self-concept as a key predictor of longitudinal change in reading achievement within ecological models that account for family, school, and sociodemographic influences. Although academic engagement, self-efficacy, and academic self-concept are closely related, they capture different dimensions of academic functioning. In the present study, academic self-concept is conceptualized as the central psychological construct, whereas engagement and self-efficacy represent complementary motivational processes.

### 1.3 Family influences on reading development

Family environments are critical for reading achievement because literacy development is embedded in everyday contexts where children encounter language, books, expectations, cultural practices, and learning opportunities. From ecological, sociocultural, and family literacy perspectives, families influence reading not only through socioeconomic resources, but also through literacy practices, parental beliefs, educational aspirations, emotional support, and the value attributed to reading (Bronfenbrenner, 1979; Claes et al., 2024; Colgate and Colgate, 2025; Dahl-Leonard et al., 2025; Yu et al., 2025). Thus, family influences should be understood as multidimensional, involving both structural resources and relational processes that shape students’ academic engagement and literacy trajectories (Gubbins and Otero, 2020; Mudrák et al., 2020; Tong et al., 2023).

The home literacy environment is commonly conceptualized as a multidimensional construct that includes active components, such as parent–child literacy activities, and passive components, such as exposure to reading models and literacy-related resources (Claes et al., 2024; Colgate and Colgate, 2025; Dahl-Leonard et al., 2025; Hayes et al., 2025). Access to books, active home reading climates, parental literacy practices, and children’s reading frequency are positively associated with reading comprehension, vocabulary, motivation, enjoyment, and subsequent reading achievement (Claes et al., 2024; Moon and Hofferth, 2016; Tong et al., 2023; Yu et al., 2025). These experiences may support reading development by increasing exposure to literacy and fostering more positive academic self-beliefs and engagement.

Socioeconomic status, parental education, and cultural capital are among the most consistent family-related predictors of reading achievement. Parental investment and cultural capital perspectives suggest that socioeconomic resources enable families to provide cognitively stimulating environments, educational support, and literacy experiences that accumulate across children’s educational trajectories (Claes et al., 2024; Colgate and Colgate, 2025; Mudrák et al., 2020; Yu et al., 2025). These resources may also shape parent–child interactions, literacy models, and the motivational meanings attached to reading and schooling (Gubbins and Otero, 2020; Ma et al., 2023; Tong et al., 2023).

Parental educational expectations are another important family factor. High expectations may shape students’ educational goals, persistence, academic orientation, and beliefs about their capacity to succeed, especially when accompanied by support and learning opportunities (Gubbins and Otero, 2020; Mudrák et al., 2020; Psyridou et al., 2025; Salvo-Garrido et al., 2023). In Chile, SIMCE-based studies have shown that parental educational expectations and perceived parental self-efficacy are positively associated with achievement, including among students attending low-SES schools (Bravo Sanzana et al., 2015; Gubbins and Otero, 2020; Salvo-Garrido et al., 2023). Family reading attitudes may also promote engagement, persistence, and favorable orientations toward literacy when families encourage reading, discuss books, model positive reading behaviors, and communicate the usefulness and enjoyment of reading (Claes et al., 2024; Gómez and Rivas, 2022; Psyridou et al., 2025; Vasalampi et al., 2023). These processes are consistent with expectancy-value approaches, which emphasize the role of perceived competence, interest, task value, and meanings communicated by significant others (Geng et al., 2023; Ma et al., 2023; Psyridou et al., 2025).

At the same time, family influences may vary across socioeconomic, cultural, gendered, and ethnic contexts. Economic pressure, limited literacy resources, family stress, and social vulnerability may constrain support for reading development, whereas warmth, cohesion, and supportive involvement may function as protective factors (Gómez and Rivas, 2022; Lam and Chung, 2023; Moon and Hofferth, 2016; Psyridou et al., 2025). In culturally diverse and Indigenous contexts, families may also transmit rich literacy practices through oral storytelling, community histories, bilingual resources, and culturally grounded knowledge that are not always recognized by standardized school expectations (Hayes et al., 2025; Ramos Salazar et al., 2023).

In Chile, family influences are particularly important because educational outcomes remain stratified by socioeconomic background, parental education, school sector, and territorial inequalities. Cultural capital, parental education, access to books, family reading attitudes, and educational expectations may contribute to unequal reading opportunities while also offering potential points for intervention (Bravo Sanzana et al., 2015; Gubbins and Otero, 2020; Salvo-Garrido et al., 2023; Yu et al., 2025). Accordingly, family-related variables are conceptualized in the present study as components of a broader ecological system that interacts with students’ psychological characteristics and school experiences to shape longitudinal change in reading achievement.

### 1.4 School climate, violence, and peer aggression

In addition to individual and family influences, school environments play a crucial role in reading development. School climate is commonly understood as a multidimensional construct that reflects the quality of school life, including norms, values, safety, academic support, belonging, classroom organization, and relationships among students, teachers, and school authorities (Su and Lee, 2023; Tan et al., 2022). From an ecological perspective, school climate constitutes a proximal context that shapes students’ daily learning experiences, socioemotional adjustment, academic engagement, and reading development (Bronfenbrenner, 1979; Dimitropoulou et al., 2025; Tan et al., 2022). Positive climates, characterized by safety, support, orderly classrooms, constructive relationships, and high academic expectations, may promote engagement and learning, whereas negative school experiences may undermine academic and socioemotional development (Ma et al., 2022; Su and Lee, 2023; Tan et al., 2022; Xiao et al., 2024).

School climate may influence reading achievement through instructional, relational, and motivational mechanisms. Orderly classrooms and positive disciplinary climates provide the structure needed for students to concentrate, participate, ask questions, and engage with increasingly complex texts (Ma et al., 2022; Tan et al., 2022; Xiao et al., 2024). Teacher support, feedback, and classroom regulation may strengthen academic self-concept, perceived competence, school belonging, and mastery-oriented motivation (Chen et al., 2021; Ma et al., 2022; Mudrák et al., 2020; Tan et al., 2022). Thus, school climate should be considered a relational and motivational context that can either facilitate or constrain reading engagement and achievement, particularly when supportive or adverse experiences accumulate over time (Dimitropoulou et al., 2025; Ren et al., 2025; Wang and Hu, 2025).

Conversely, school violence, bullying, and peer aggression may function as chronic stressors that interfere with learning. Exposure to aggressive interactions, victimization, threats, or disruptive classrooms may reduce students’ sense of safety, emotional security, concentration, perceived control, and willingness to participate in school activities (Bravo-Sanzana et al., 2022; Salvo-Garrido et al., 2020; Su and Lee, 2023). The development of reading skills is supported by sustained attention, confidence, motivation, and active engagement with texts; therefore, hostile school and peer environments may undermine the cognitive and socioemotional conditions needed to develop literacy skills over time (Dimitropoulou et al., 2025; Tan et al., 2022; Wang and Hu, 2025). Previous Chilean evidence has similarly shown that school violence, bullying, and discrimination are negatively associated with standardized academic performance in Reading, Mathematics, and other curricular domains (Bravo-Sanzana et al., 2022; Salvo-Garrido et al., 2020; Salvo-Garrido et al., 2023).

Discriminatory school climates may also affect academic development by weakening students’ sense of belonging, emotional security, perceived fairness, and trust in the school environment. Cross-national evidence using PISA 2018 data shows that perceived discriminatory climate is associated with lower mathematics and reading performance among both ethnic majority and minority students, with school belonging and the value attributed to learning partially mediating these associations (Baysu et al., 2023; Heppt et al., 2025). This is particularly relevant for studies examining Indigenous status, as culturally or ethnically minoritized students may experience school environments that insufficiently recognize their identities, languages, histories, territories, and community-based knowledge (Arias-Ortega and Villarroel-Cárdenas, 2024; Arias Ortega and Del Pino, 2025; Torres Cuevas et al., 2024).

Teacher–student relationships are another important dimension of school climate. Supportive relationships may foster academic confidence, engagement, help-seeking, and persistence, whereas conflictive interactions or student–teacher aggression may contribute to disengagement and less favorable academic outcomes (Bravo-Sanzana et al., 2022; Ma et al., 2022; Salvo-Garrido et al., 2020; Tang et al., 2022). In reading, students often require feedback, scaffolding, encouragement, and opportunities to discuss texts in order to develop comprehension and critical interpretation skills (Chen et al., 2021; Ma et al., 2022; Ohle-Peters et al., 2023; Preusler et al., 2022). Accordingly, classroom relationships represent not only instructional resources but also developmental contexts in which students construct confidence, persistence, and positive academic self-perceptions (Dimitropoulou et al., 2025; Ma et al., 2022; Tan et al., 2022; Wang and Hu, 2025).

Overall, school climate and aggression-related factors should be considered central components of reading development. Safe, inclusive, and well-organized schools may provide the relational and instructional conditions necessary for students to develop confidence, engagement, and persistence in reading, whereas violence, peer aggression, discrimination, and conflictive student–teacher interactions may weaken learning opportunities over time (Baysu et al., 2023; Bravo-Sanzana et al., 2022; Heppt et al., 2025; Salvo-Garrido et al., 2023; Xiao et al., 2024). Accordingly, school-related variables are conceptualized in the present study as ecological conditions that, in interaction with students’ individual characteristics and family environments, shape longitudinal change in reading achievement between primary and lower secondary education.

### 1.5 The present study: research questions and hypotheses

Although previous research has identified multiple factors associated with reading achievement, important gaps remain. Much evidence is cross-sectional, limiting understanding of how reading performance changes across schooling and how early individual, family, and school conditions are associated with later achievement (Claes et al., 2024; Larsen et al., 2022; Maclean et al., 2024; Tang et al., 2022). Psychological, family, and school predictors have also often been examined separately, despite evidence that reading development is shaped by interactions among self-beliefs, family literacy resources, instructional experiences, school climate, and socioeconomic conditions (Bronfenbrenner, 1979; Chen et al., 2021; Ma et al., 2023; Mudrák et al., 2020; Yu et al., 2025). Longitudinal studies using large-scale national data to examine longitudinal change in reading achievement by student-reported sex, Indigenous status, and socioeconomic status remain scarce in Latin America, as do studies integrating psychological, family, school, and sociodemographic dimensions within an ecological framework (Bravo-Sanzana et al., 2022; Cubillos and Troncoso, 2025; Gubbins and Otero, 2020; Salvo-Garrido et al., 2023).

Chile provides a relevant context for addressing these gaps because its standardized assessment system coexists with persistent educational inequalities associated with socioeconomic background, school segregation, territorial disparities, gendered performance patterns, and ethnic origin (Bravo Sanzana et al., 2015; Cubillos and Troncoso, 2025; Gubbins and Otero, 2020; Salvo-Garrido et al., 2023). Chilean evidence has linked academic outcomes to self-efficacy, parental expectations, school violence, discrimination, teacher-related factors, and socioeconomic resources, but fewer studies have examined how these factors jointly relate to longitudinal change in reading achievement between primary and lower secondary education (Bravo-Sanzana et al., 2022; Gubbins and Otero, 2020; Salvo-Garrido et al., 2020; Salvo-Garrido et al., 2023). This joint approach is especially relevant from an equity perspective because reading achievement may vary by sex-related motivational, attitudinal, strategic, and sociocultural factors (Acar-Erdol and Akin-Arikan, 2022; Reinhold et al., 2026; Steinmann and Strietholt, 2026; Taraszow et al., 2024), by the extent to which Indigenous and culturally minoritized students’ identities, languages, territories, histories, and community knowledge are recognized at school (Arias-Ortega and Villarroel-Cárdenas, 2024; Arias Ortega and Del Pino, 2025; Torres Cuevas et al., 2024), and by socioeconomic access to cultural capital, books, family literacy practices, parental education, and school resources (Claes et al., 2024; Colgate and Colgate, 2025; Ma et al., 2023; Yu et al., 2025).

The current study examines longitudinal change in reading achievement among Chilean students between Grade 4 and Grade 8 using a national longitudinal dataset and an ecological framework that integrates psychological, family, school, and sociodemographic predictors of reading development. Drawing on educational psychology, ecological theory, family literacy research, and school climate perspectives, it analyzes whether Grade 4 predictors are associated with subsequent reading achievement, with particular attention to academic self-concept, academic engagement and self-efficacy, teacher regulation and normative guidance, family reading attitudes, parental education, cultural capital, parental educational expectations, school climate, violence, peer aggression, perceived discrimination, student-reported sex, Indigenous status, and socioeconomic status (Basarkod et al., 2023; Bronfenbrenner, 1979; Chen et al., 2021; Claes et al., 2024; Ma et al., 2022; Salvo-Garrido et al., 2023). These variables are treated as complementary components of the developmental ecology surrounding reading achievement.

Guided by this framework, the study addressed five research questions concerning: changes in reading achievement between Grade 4 and Grade 8; the contribution of individual factors; the role of family-related factors; the association of school climate, violence, peer aggression, perceived discrimination, and student–teacher aggression with reading development; and whether longitudinal change differed by student-reported sex, Indigenous status, and socioeconomic status. We expected reading achievement to differ between Grade 4 and Grade 8; positive individual and family factors to be positively associated with reading achievement; negative school experiences to be negatively associated with achievement; and longitudinal change to differ by student-reported sex, Indigenous status, and socioeconomic status.

## 2 Materials and methods

### 2.1 Study design and data source

This study employed a longitudinal observational design based on secondary data from a national cohort of Chilean students who participated in the National System for the Assessment of Educational Quality (SIMCE). SIMCE is Chile’s national large-scale assessment system for monitoring student achievement in key curricular areas and collecting contextual information from students, families, teachers, and schools through complementary questionnaires (Bravo Sanzana et al., 2015; Salvo-Garrido et al., 2023).

The study followed students in two specific grades: Grade 4 in 2015 and Grade 8 in 2019. Reading achievement scores from both assessment cycles were linked at the student level using unique anonymized identifiers, allowing repeated achievement measures to be organized longitudinally. Baseline predictors were obtained from student, parent/guardian, and teacher questionnaires administered in Grade 4. This design made it possible to examine how individual, family, school-related, and sociodemographic factors measured at baseline were associated with longitudinal change in reading achievement across 4 years.

The study was based on observational secondary data, and the analyses were designed to identify longitudinal associations rather than causal effects. All achievement and questionnaire records were provided in anonymized form by the Chilean Education Quality Assurance Agency under confidentiality conditions, and no personally identifiable information was available to the researchers.

### 2.2 Participants

The analytical sample included 123,827 students who completed the SIMCE Reading assessment in Grade 4 in 2015 and were successfully followed through the Grade 8 Reading assessment in 2019. Students were included if they had complete information on reading achievement at both measurement occasions and on the baseline predictors retained for the longitudinal models.

The sample comprised 64,314 girls (51.9%) and 59,513 boys (48.1%). Indigenous status was derived from the Grade 4 parent/guardian questionnaire; 18,995 students (15.3%) were reported as belonging to an Indigenous group, whereas 104,832 students (84.7%) were reported as having no Indigenous affiliation. Regarding school sector, 71,061 students (57.4%) attended subsidized private schools, 41,815 (33.8%) attended public schools, and 10,951 (8.8%) attended private paid schools. Most students were enrolled in urban schools (*n* = 111,244; 89.9%), while 12,583 students (10.2%) attended rural schools.

To assess potential selection associated with longitudinal attrition and complete-case inclusion, we conducted sensitivity analyses comparing students retained through Grade 8 with those lost to follow-up, and, among longitudinally retained students, those included in the final analytic sample with those excluded because of incomplete analytic data. Comparisons included baseline reading achievement, sociodemographic characteristics, and the continuous questionnaire-based predictors included in the final model. Results of these analyses are reported in Section 3.1.

### 2.3 Measures

Reading achievement was assessed using students’ scores on the SIMCE Reading tests administered in Grade 4 in 2015 and Grade 8 in 2019. SIMCE is Chile’s national assessment system for monitoring student learning outcomes in key curricular areas and collecting contextual information from students, families, teachers, and schools through complementary questionnaires (Bravo Sanzana et al., 2015; Salvo-Garrido et al., 2023). Reading achievement scores from both assessment cycles were used as the repeated outcome measure in the longitudinal analyses; thus, each student contributed two reading achievement observations.

Individual factors were measured using indices derived from the Grade 4 student questionnaire. Five student-level predictors were included: academic engagement and self-efficacy, academic self-concept, teacher regulation and normative guidance, school safety perception, and school violence and peer aggression. Academic engagement and self-efficacy captured students’ motivation, effort, persistence, and confidence in their academic abilities. Academic self-concept measured students’ perceptions of their own academic competence. Teacher regulation and normative guidance assessed students’ perceptions of teachers’ guidance, expectations, feedback, and support for classroom behavior and learning. School safety perception captured students’ sense of safety within the school environment, whereas school violence and peer aggression assessed students’ experiences and perceptions of aggressive behaviors, conflicts, and violence among peers. Higher scores indicated higher levels of the corresponding construct.

Family-related factors were measured using information from the Grade 4 parent/guardian questionnaire. Five family-level predictors were included: parental education, cultural capital, parental educational expectations, family reading attitudes, and perceived discrimination at school. Parental education represented the educational attainment of students’ parents or guardians. Cultural capital was operationalized through the number of books available at home. Parental educational expectations reflected families’ educational aspirations for their children. Family reading attitudes measured the extent to which reading was valued, encouraged, and promoted within the home environment. Perceived discrimination at school assessed family reports of discriminatory experiences affecting students in school settings.

School-related factors were derived from the Grade 4 teacher questionnaires. Three school-level indicators were included: classroom order and engagement, school violence climate, and student–teacher aggression. Classroom order and engagement evaluated teachers’ perceptions of classroom organization, student participation, and engagement in learning activities. School violence climate measured the perceived prevalence of violent behaviors, conflicts, and disciplinary problems within the school environment. Student–teacher aggression assessed the frequency of verbal and physical aggressive interactions involving students and teachers. Higher scores on classroom order and engagement reflected more favorable classroom conditions, whereas higher scores on school violence climate and student–teacher aggression reflected more adverse school environments.

Sex was measured through the Grade 4 student questionnaire and included as a categorical predictor, with girls as the reference category. The variable included binary categories for boys and girls and is therefore interpreted as sex as recorded in the SIMCE database, rather than as a measure of gender identity. Indigenous status was derived from the Grade 4 parent/guardian questionnaire and included as a categorical predictor, with non-Indigenous students as the reference category. Socioeconomic status was based on household information reported in the parent/guardian questionnaire and represented by a five-level index derived from household socioeconomic characteristics. School sector distinguished public, subsidized private, and private paid schools, with private paid schools used as the reference category. School habitat distinguished urban and rural schools, with rural schools used as the reference category.

A detailed description of the questionnaire-based measures, including domain, questionnaire source, scale type, number of items, internal consistency estimates where applicable, and interpretation, is provided in Supplementary Table S1.

### 2.4 Index construction and predictor selection

Questionnaire-based indices were constructed from the student, parent/guardian, and teacher questionnaires administered in Grade 4. Each index was calculated as the arithmetic mean of the items comprising the corresponding construct, preserving the original response scale. Higher scores indicated higher levels of the construct; for positively oriented indices, higher values reflected more favorable conditions, whereas for risk-related indices, higher values reflected more adverse conditions.

The selection of candidate predictors was guided by the theoretical framework developed in the Introduction, which identified individual, family, school, and sociodemographic factors as key dimensions associated with reading achievement and its longitudinal change. Within this theoretically defined set of variables, a Random Forest procedure was subsequently employed as an exploratory data-driven strategy to identify the most informative questionnaire-based indices for inclusion in the inferential analyses.

Initially, questionnaire-derived indices available from the Grade 4 student, parent/guardian, and teacher contextual questionnaires were screened separately within each source domain using Random Forest models. This domain-specific approach was used to identify the indices showing the greatest empirical relevance within the student, family, and teacher-related sets of predictors, while preserving the ecological structure underlying the study.

Random Forests are useful for exploratory variable selection because they can handle multiple predictors, capture nonlinear patterns, and provide variable-importance measures (Breiman, 2001; Genuer et al., 2010). Consistent with previous SIMCE-based research, the procedure was used exclusively as an exploratory screening tool prior to inferential modeling (Bravo Sanzana et al., 2015). Accordingly, the Random Forest analysis was not interpreted as inferential evidence, nor was it used to draw substantive conclusions regarding the relative importance or causal role of the retained predictors.

The Random Forest models were estimated using 1,000 trees, with three predictors randomly considered at each split, a minimum split size of 50 observations, and a fixed random seed of 2026. Variable importance was assessed using the Column Contributions measure, which quantifies the relative contribution of each predictor to the reduction in residual sum of squares across the ensemble.

Because variable-importance measures do not provide causal or confirmatory evidence and may be affected by correlations among predictors or scale differences (Strobl et al., 2007), the final selection of predictors was based on both empirical relevance and theoretical relevance to reading development, family literacy, and school climate research. All retained predictors were measured at baseline in Grade 4 and used to examine longitudinal associations with reading achievement between Grade 4 and Grade 8.

A detailed description of the questionnaire-based indices that were significant in the final reading achievement model is provided in Supplementary Table S1. The table reports construct labels, domain, questionnaire source, scale type, number of items, internal consistency estimates where applicable, and interpretation, without reproducing item codes or item wording due to data-use restrictions.

### 2.5 Procedure

Student achievement records from the Grade 4 SIMCE Reading assessment administered in 2015 were linked to the corresponding Grade 8 Reading assessment conducted in 2019 using unique anonymized student identifiers. This linkage made it possible to follow the same students across both assessment cycles and to examine change in reading achievement over a four-year period.

Contextual information from student, parent/guardian, and teacher questionnaires administered in Grade 4 was incorporated as baseline information. These questionnaires provided individual, family, school-related, and sociodemographic predictors hypothesized to be associated with reading achievement over time.

The final dataset was structured in long format, with repeated reading achievement measures nested within students. Two measurement occasions were considered: Grade 4 in 2015 and Grade 8 in 2019. All predictors were measured at baseline in Grade 4.

All analyses were conducted using anonymized secondary data provided by the Chilean Education Quality Assurance Agency under confidentiality conditions. The research team had no access to personally identifiable information, and no direct contact with students, families, teachers, or schools was involved.

### 2.6 Data analysis

Longitudinal change in reading achievement was analyzed using linear mixed-effects models (LMMs) with repeated measures. This approach was appropriate because each student contributed two reading achievement scores, one in Grade 4 and one in Grade 8, and the observations were therefore not independent. The models accounted for the dependency of repeated measures within students while estimating both within-student change over time and between-student differences in reading achievement (Raudenbush and Bryk, 2002; Singer and Willett, 2003). Given the two-occasion longitudinal design, a random-intercept specification was selected as the most parsimonious structure for capturing between-student heterogeneity while estimating average change over time.

$$Y_{\mathrm{it}}=\beta_{0}+\beta_{1}\mathrm{Tim}e_{\mathrm{it}}+\sum_{k=1}^{p}\beta_{k}X_{\mathrm{ki}}+\sum_{m=1}^{q}\beta_{m}Z_{\mathrm{mi}}+u_{i}+\varepsilon_{\mathrm{it}.}$$

Where $Y_{\mathrm{it}}$ represents the reading achievement score of student *i* at measurement occasion *t*; $\beta_{0}$ is the fixed intercept; $\beta_{1}$ represents the fixed effect of time; $X_{\mathrm{ki}}$ denotes the baseline individual, family, school-related, and contextual predictors measured in Grade 4; $Z_{\mathrm{mi}}$ denotes the interaction terms included in the moderation model; $\beta_{k}$ and $\beta_{m}$ represent the fixed-effect coefficients associated with the baseline predictors and interaction terms, respectively; $u_{i}$ is the student-specific random intercept capturing unobserved between-student variability; and $\varepsilon_{\mathrm{it}}$ is the residual error term.

The analytical strategy proceeded in four steps. First, an unconditional null model was estimated to examine variability in reading achievement before adding predictors. Second, an integrated model included time, student-reported sex, Indigenous status, socioeconomic status, school sector, school habitat, and all individual, family, and school-related predictors measured in Grade 4. Third, interaction models were estimated to test whether longitudinal change in reading achievement differed according to student-reported sex, Indigenous status, and socioeconomic status. Finally, higher-order interaction models were estimated, including the Time × Sex × Indigenous Status model and the Time × Sex × Indigenous Status × Socioeconomic Status model.

Model comparisons were conducted using maximum likelihood estimation because the models differed in their fixed-effect structure. Model fit was evaluated using −2 log-likelihood, Akaike Information Criterion (AIC), and Bayesian Information Criterion (BIC), with lower values indicating better fit. Once the best-fitting and most parsimonious model was identified, final parameter estimates were obtained using restricted maximum likelihood estimation.

Random intercepts were specified at the student level to account for the dependency between repeated measurements. Statistical significance was evaluated at the 0.05 level using the Satterthwaite approximation for degrees of freedom. Estimated marginal means from the final model were used to graphically represent model-based change in reading achievement and to support the interpretation of interaction effects.

Data management and restructuring were conducted in R using the *tidyr* and *dplyr* packages. Linear mixed-effects models were estimated using the *lmerTest* package, which provides tests of fixed effects based on Satterthwaite’s approximation for degrees of freedom. Estimated marginal means and model-based contrasts used for the interpretation of interactions and graphical representation of longitudinal change were obtained with the *emmeans* package.

Because the study used observational secondary data, the estimated associations should not be interpreted as causal effects. Rather, the models identify baseline individual, family, school, and contextual factors associated with reading achievement across the four-year longitudinal period.

Potential selection associated with longitudinal attrition and complete-case inclusion was evaluated in two stages. First, students with valid Grade 4 reading scores who were retained through Grade 8 were compared with those lost to follow-up on baseline reading achievement and sociodemographic characteristics (sex, Indigenous status, socioeconomic status, school sector, and habitat). Second, among students with reading scores at both assessment occasions, those included in the final analytic sample were compared with those excluded because of incomplete covariate data. These comparisons included baseline reading achievement, sociodemographic characteristics, and all continuous questionnaire-based predictors included in the final model. Cohen’s d was used for continuous variables and Cramér’s V for categorical variables to emphasize the magnitude of group differences rather than statistical significance alone.

## 3 Results

### 3.1 Descriptive statistics

The final longitudinal sample included 123,827 students who participated in both the Grade 4 (2015) and Grade 8 (2019) SIMCE Reading assessments. Descriptive statistics for reading achievement, baseline predictors, and sample characteristics are presented in Table 1. Reading achievement was measured at both time points, whereas all individual, family, school-related, and sociodemographic predictors were measured at baseline in Grade 4.

**Table 1:** Descriptive statistics and sample characteristics (*N* = 123,827).

Variable	M	SD	Min	Max
Reading achievement (grade 4)	273.0	48.06	118.0	389.0
Reading achievement (grade 8)	247.3	50.01	102.7	364.8
Academic engagement and self-efficacy	3.11	0.39	1.00	4.00
Academic self-concept	2.92	0.53	1.00	4.00
Teacher regulation and normative guidance	3.50	0.53	1.00	4.00
School safety perception	3.03	0.83	1.00	4.00
School violence and peer aggression	2.00	0.70	1.00	4.00
Parental education	2.39	0.93	1.00	5.00
Cultural capital (books at home)	3.04	0.99	1.00	5.00
Parental educational expectations	4.90	0.98	1.00	6.00
Family reading attitudes	2.94	0.61	1.00	4.00
Perceived discrimination at school	1.20	0.37	1.00	4.00
Classroom order and engagement	3.48	0.36	1.00	4.00
School violence climate	1.68	0.49	1.00	4.00
Student–teacher aggression	1.17	0.31	1.00	4.00

Sample characteristics	*n*	%
Boys	59,513	48.1
Girls	64,314	51.9
Indigenous students	18,995	15.3
Non-Indigenous students	104,832	84.7
Public schools	41,815	33.8
Subsidized private schools	71,061	57.4
Private paid schools	10,951	8.8
Urban schools	111,244	89.9
Rural schools	12,583	10.2

Reading achievement scores correspond to SIMCE Reading assessments in Grade 4 (2015) and Grade 8 (2019). All questionnaire-based predictors and sociodemographic characteristics were measured at baseline in Grade 4. Higher scores on questionnaire-based indices indicate higher levels of the corresponding construct.

Observed reading scores were lower at the Grade 8 assessment occasion (M = 247.3, SD = 50.01) than at the Grade 4 assessment occasion (M = 273.0, SD = 48.06), indicating lower observed performance in the second SIMCE Reading assessment. The sample was relatively balanced by student-reported sex, with girls representing 51.9% of participants and boys 48.1%. Indigenous students accounted for 15.3% of the sample. Most students attended subsidized private schools (57.4%) and urban schools (89.9%). Baseline questionnaire indices showed variability across individual, family, and school-related domains, supporting their inclusion in the longitudinal model.

The eligible Grade 4 cohort comprised 207,733 students. Of these, 168,162 had valid reading scores at both assessment occasions, corresponding to a longitudinal retention rate of 80.95% and an attrition rate of 19.05%. Students retained through Grade 8 had higher Grade 4 reading achievement than those lost to follow-up (M = 270.56 vs. 243.73; Cohen’s *d* = 0.55), indicating a moderate baseline achievement difference. Differences in baseline sociodemographic characteristics were considerably smaller (Cramér’s V = 0.011–0.082). Among the 168,162 longitudinally retained students, 123,827 (73.64%) were included in the final analytic sample and 44,335 (26.36%) were excluded because of incomplete analytic data. The corresponding difference in Grade 4 reading achievement was small (M = 273.02 vs. 263.68; *d* = 0.19), as were differences in sociodemographic characteristics (Cramér’s V = 0.007–0.052) and in the continuous questionnaire-based predictors included in the final model (|*d*| = 0.064–0.152). These findings indicate that selection into the final analytic sample was generally small with respect to observed covariates, although longitudinal attrition was associated with a moderate difference in baseline reading achievement.

### 3.2 Model comparison and final model selection

Model comparisons were conducted using maximum likelihood estimation and are presented in Table 2. Relative to the null model, the integrated model showed a substantial improvement in fit, indicating that the inclusion of individual, family, school-related, and contextual predictors provided a better representation of longitudinal change in reading achievement.

**Table 2:** Model fit statistics for linear mixed-effects models of reading achievement.

Model	-2LL	AIC	BIC
Null model	2602539.75	2602549.75	2602601.85
Integrated model	2514437.01	2514487.01	2514747.50
Time × sex	2514097.03	2514149.03	2514419.94
Time × Indigenous status	2514426.65	2514478.65	2514749.56
Time × socioeconomic status	2514436.86	2514488.86	2514759.78
Time × sex × Indigenous status	2514078.04	2514136.04	2514438.22
Time × sex × Indigenous status × socioeconomic status	2514064.73	2514136.73	2514511.84

Model comparisons were conducted using maximum likelihood estimation. Lower values of −2 log-likelihood, Akaike Information Criterion (AIC), and Bayesian Information Criterion (BIC) indicate better model fit. The integrated model included time, student-reported sex, Indigenous status, socioeconomic status, school sector, school habitat, and all individual, family, and school predictors measured in Grade 4. Socioeconomic status was included as an ordinal covariate. Although the four-way interaction model showed the lowest −2 log-likelihood, the three-way and four-way models showed virtually equivalent AIC values, whereas BIC clearly favored the more parsimonious Time × Sex × Indigenous Status model. This model was therefore retained as the final model on grounds of parsimony.

Among the moderation models, the Time × Sex × Indigenous Status model was retained as the final and most parsimonious model. The three-way and four-way models showed virtually equivalent AIC values (ΔAIC = 0.69), indicating no meaningful difference in fit according to this criterion. In contrast, BIC clearly favored the three-way model, with a difference of approximately 73.6 points. Although the four-way model including socioeconomic status showed a slightly lower −2 log-likelihood, there was no compelling evidence that adding socioeconomic status as an additional moderator improved model fit sufficiently to justify the increased complexity. Therefore, the Time × Sex × Indigenous Status model was retained on grounds of parsimony. This result suggests that longitudinal differences in reading achievement were better explained by the joint combination of time, Sex, and Indigenous status than by adding socioeconomic status as an additional moderator of change.

### 3.3 Final mixed-effects model predicting longitudinal change in reading achievement

Final parameter estimates were obtained using restricted maximum likelihood estimation for the selected Time × Sex × Indigenous Status model. Type III tests of fixed effects were used to evaluate the omnibus significance of the fixed effects and interaction terms, whereas Table 3 presents the fixed-effect estimates from the final REML model, including the main effects and all lower- and higher-order interaction terms relevant to the retained Time × Sex × Indigenous Status structure. The retained model included all lower-order terms required by the interaction hierarchy.

**Table 3:** Fixed-effect estimates from the final linear mixed-effects model predicting longitudinal change in reading achievement (*N* = 123,827).

Predictor	B	SE	df	*t*	*p*	95% CI
Intercept	145.05	2.09	124152.6	69.40	<0.001	[141.0, 149.1]
Time (Grade 8)	−23.68	0.17	126448.0	−135.95	<0.001	[−24.0, −23.3]
Boys	−4.39	0.25	163672.0	−17.39	<0.001	[−4.9, −3.9]
Indigenous status	1.44	0.45	163817.3	3.22	0.001	[0.6, 2.3]
Public school	−10.05	0.55	123869.2	−18.37	<0.001	[−11.1, −9.0]
Subsidized private school	−5.14	0.48	123869.2	−10.65	<0.001	[−6.1, −4.2]
Urban	−1.43	0.38	123869.2	−3.81	<0.001	[−2.2, −0.7]
Socioeconomic status (SES)	1.76	0.14	123869.2	12.73	<0.001	[1.5, 2.0]
Time × boys	−4.48	0.25	126446.1	−17.85	<0.001	[−5.0, −4.0]
Time × indigenous status	0.33	0.44	123823.0	0.75	0.456	[−0.5, 1.2]
Boys × indigenous status	−1.71	0.64	164424.4	−2.67	0.008	[−3.0, −0.5]
Time × boys × indigenous status	1.44	0.64	126448.1	2.25	0.025	[0.2, 2.7]
Individual factors (students)						
Academic engagement and self-efficacy	2.75	0.31	123869.2	8.99	<0.001	[2.1, 3.3]
Academic self-concept	23.45	0.22	123869.2	106.06	<0.001	[23.0, 23.9]
Teacher regulation and normative guidance	7.91	0.21	123869.2	37.41	<0.001	[7.5, 8.3]
School safety perception	0.82	0.14	123869.2	5.74	<0.001	[0.5, 1.1]
School violence and peer aggression	−10.15	0.17	123869.2	−59.14	<0.001	[−10.5, −9.8]
Family factors (parents/guardians)						
Parental education	4.37	0.18	123869.2	24.92	<0.001	[4.0, 4.7]
Cultural capital (books at home)	2.07	0.12	123869.2	16.59	<0.001	[1.8, 2.3]
Parental educational expectations	4.65	0.13	123869.2	36.86	<0.001	[4.4, 4.9]
Family reading attitudes	2.28	0.19	123869.2	12.24	<0.001	[1.9, 2.6]
Perceived discrimination at school	−6.48	0.30	123869.2	−21.85	<0.001	[−7.1, −5.9]
School factors (teacher)						
Classroom order and engagement	4.12	0.34	123869.2	12.05	<0.001	[3.4, 4.8]
School violence climate	−1.51	0.29	123869.2	−5.29	<0.001	[−2.1, −0.9]
Student–teacher aggression	−2.54	0.44	123869.2	−5.83	<0.001	[−3.4, −1.7]

The final model was estimated using restricted maximum likelihood (REML). B, unstandardized estimated coefficient; SE, standard error; df, Satterthwaite degrees of freedom; CI, confidence interval. Positive coefficients indicate higher estimated reading achievement scores, whereas negative coefficients indicate lower estimated reading achievement scores. Grade 4 was the reference category for Time. Reference categories were girls, non-Indigenous students, rural schools, and private paid schools. All questionnaire-based predictors were measured at baseline in Grade 4. For terms involved in interactions, coefficients are conditional contrasts relative to the reference categories. The table reports the main fixed-effect estimates and all lower- and higher-order interaction terms relevant to the retained Time × Sex × Indigenous Status structure. Type III tests reported in the text provide omnibus tests of fixed effects and interaction terms.

Type III tests of fixed effects supported the selected moderation structure. The highest-order interaction, Time × Sex × Indigenous Status, was statistically significant, *F*(1, 126448.068) = 5.051, *p* = 0.025, indicating that longitudinal change in reading achievement differed according to the joint combination of assessment occasion, student-reported sex, and Indigenous status. Significant lower-order interactions were also observed for Time × Sex, *F*(1, 126448.068) = 137.725, *p* < 0.001, and Time × Indigenous Status, *F*(1, 126448.068) = 10.756, *p* = 0.001. The Sex × Indigenous Status interaction was not statistically significant in the Type III tests, *F*(1, 126526.339) = 2.820, *p* = 0.093, but was retained to preserve the hierarchical structure of the model.

As shown in Table 3, because the final model retained a Time × Sex × Indigenous Status interaction, the coefficients for time, boys, Indigenous status, and the selected interaction terms should be interpreted as conditional contrasts relative to the reference categories rather than as average effects for the entire sample. The Grade 8 assessment indicator showed a negative coefficient relative to Grade 4, indicating lower observed reading performance at the second assessment occasion (B = −23.68, SE = 0.17, *p* < 0.001). This coefficient should be interpreted as a difference between SIMCE assessment occasions rather than as direct evidence of a causal decline in reading ability. The negative Time × Boys coefficient (B = −4.48, SE = 0.25, *p* < 0.001) indicates that, among non-Indigenous students, the model-based difference between the Grade 4 and Grade 8 assessment occasions was larger for boys than for girls. The significant Time × Boys × Indigenous Status interaction (*B* = 1.44, SE = 0.64, *p* = 0.025) indicates that the sex difference in model-based change across assessment occasions varied by Indigenous status. Accordingly, subgroup-specific patterns of change are interpreted primarily through the estimated marginal means presented in Figure 1. Socioeconomic status, which was not part of the retained moderation structure, was positively associated with reading achievement (*B* = 1.76, SE = 0.14, *p* < 0.001), indicating higher estimated reading scores among students from more advantaged socioeconomic backgrounds.

**Figure 1:**
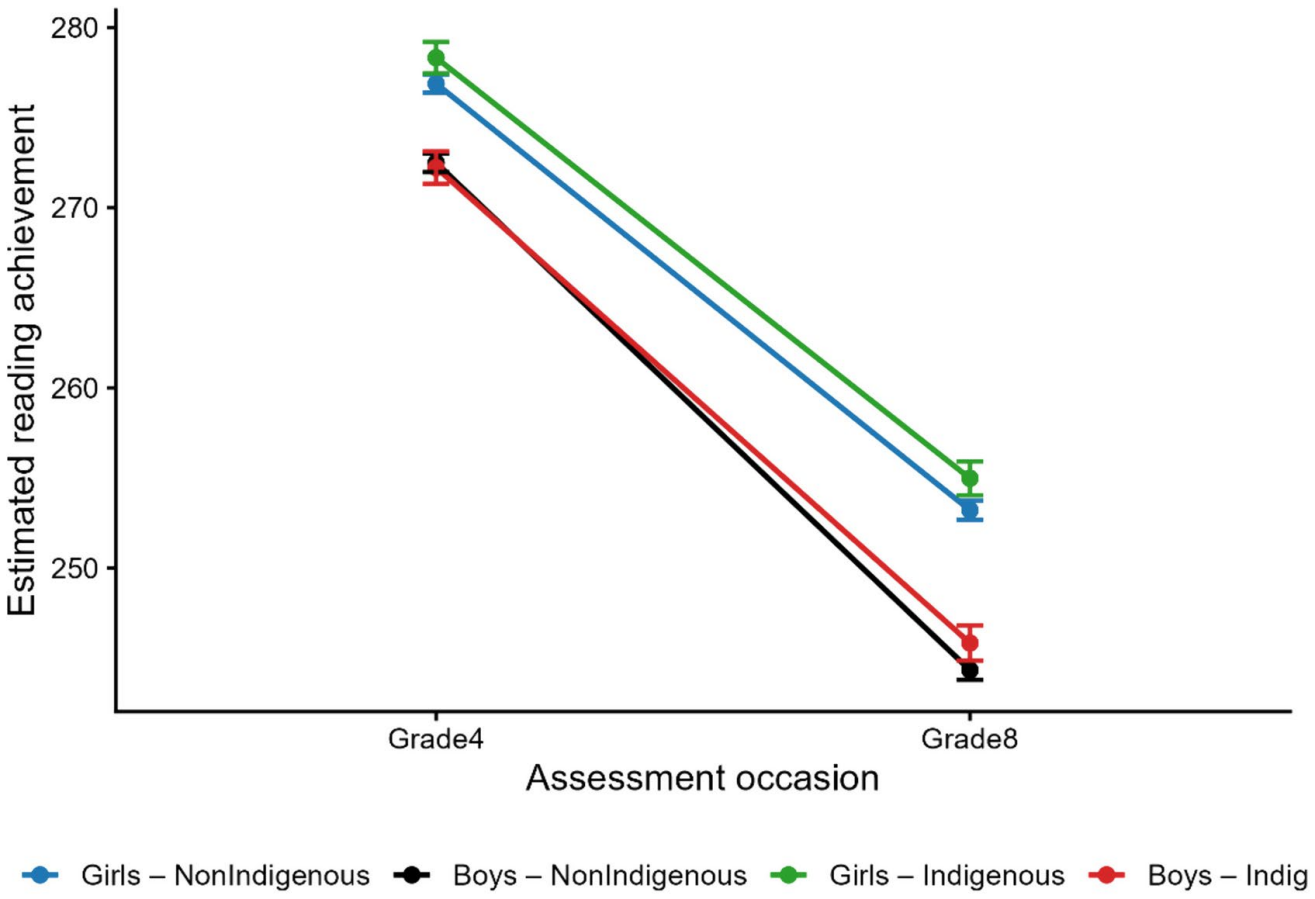
Estimated reading achievement change from Grade 4 (2015) to Grade 8 (2019) by student-reported sex and Indigenous status. Estimated marginal means were derived from the final linear mixed-effects model including the Time × Sex × Indigenous Status interaction. Error bars represent 95% confidence intervals. Sex was reported by students in the Grade 4 student questionnaire, whereas Indigenous status was derived from the Grade 4 parent/guardian questionnaire.

Given the large sample size, standardized effect magnitudes were also considered to aid substantive interpretation. The conditional effect associated with Indigenous status was small (*B* = 1.44, approximately 0.03 SD), as was the urban–rural contrast (*B* = −1.43, approximately −0.03 SD). Likewise, the significant Time × Sex × Indigenous Status interaction was small in standardized magnitude (*B* = 1.44, approximately 0.03 SD), indicating that statistical significance should not be interpreted as evidence of a large practical effect.

Among individual factors, academic self-concept showed a strong positive association with reading achievement (*B* = 23.45, SE = 0.22, *p* < 0.001). Teacher regulation and normative guidance also showed a positive association (*B* = 7.91, SE = 0.21, *p* < 0.001), followed by academic engagement and self-efficacy (*B* = 2.75, SE = 0.31, *p* < 0.001) and school safety perception (*B* = 0.82, SE = 0.14, *p* < 0.001). In contrast, the school violence and peer aggression index was strongly and negatively associated with reading achievement (*B* = −10.15, SE = 0.17, *p* < 0.001), indicating that adverse peer and school experiences in Grade 4 were linked to lower reading performance across assessment occasions.

Family-related predictors also contributed to reading achievement. Parental educational expectations (*B* = 4.65, SE = 0.13, *p* < 0.001), parental education (*B* = 4.37, SE = 0.18, *p* < 0.001), family reading attitudes (*B* = 2.28, SE = 0.19, *p* < 0.001), and cultural capital (*B* = 2.07, SE = 0.12, *p* < 0.001) were positively associated with reading scores. Conversely, perceived discrimination at school was negatively associated with achievement (*B* = −6.48, SE = 0.30, *p* < 0.001). Regarding school-related predictors, classroom order and engagement was positively associated with reading achievement (*B* = 4.12, SE = 0.34, *p* < 0.001), whereas school violence climate (*B* = −1.51, SE = 0.29, *p* < 0.001) and student–teacher aggression (*B* = −2.54, SE = 0.44, *p* < 0.001) were negatively associated with reading performance.

Figure 1 illustrates the estimated reading achievement change by sex and Indigenous status. Model-based estimated reading scores were lower at the Grade 8 assessment occasion than at the Grade 4 assessment occasion for all groups, although the magnitude of this difference varied across subgroups. Non-Indigenous boys showed estimated scores of 272.49 (95% CI [271.98, 273.00]) in Grade 4 and 244.33 (95% CI [243.80, 244.87]) in Grade 8, whereas Indigenous boys showed estimated scores of 272.22 (95% CI [271.32, 273.13]) and 245.84 (95% CI [244.86, 246.81]), respectively. Among girls, the model-based difference was smaller: non-Indigenous girls showed estimated scores of 276.89 (95% CI [276.38, 277.39]) in Grade 4 and 253.21 (95% CI [252.68, 253.74]) in Grade 8, whereas Indigenous girls showed estimated scores of 278.32 (95% CI [277.45, 279.20]) and 254.98 (95% CI [254.04, 255.92]).

Across both measurement occasions, girls showed higher estimated reading achievement than boys, regardless of Indigenous status. Indigenous girls displayed the highest estimated scores at both time points, followed by non-Indigenous girls. The two groups of boys showed lower estimated achievement levels, although Indigenous boys had slightly higher estimated scores than non-Indigenous boys in Grade 8. This pattern is consistent with the significant Time × Sex × Indigenous Status interaction.

Figure 2 presents estimated reading achievement change for students in the lowest and highest socioeconomic groups. Students in SES 5 showed consistently higher estimated reading achievement than students in SES 1 at both assessment occasions. For example, among non-Indigenous boys, estimated scores were 270.89 (95% CI [270.28, 271.51]) in Grade 4 and 242.73 (95% CI [242.10, 243.37]) in Grade 8 for SES 1, compared with 277.92 (95% CI [277.04, 278.81]) and 249.77 (95% CI [248.87, 250.66]) for SES 5. Among non-Indigenous girls, the corresponding estimates were 275.29 (95% CI [274.68, 275.89]) and 251.61 (95% CI [250.99, 252.24]) for SES 1, and 282.32 (95% CI [281.43, 283.21]) and 258.64 (95% CI [257.74, 259.54]) for SES 5. A similar pattern was observed among Indigenous students.

**Figure 2:**
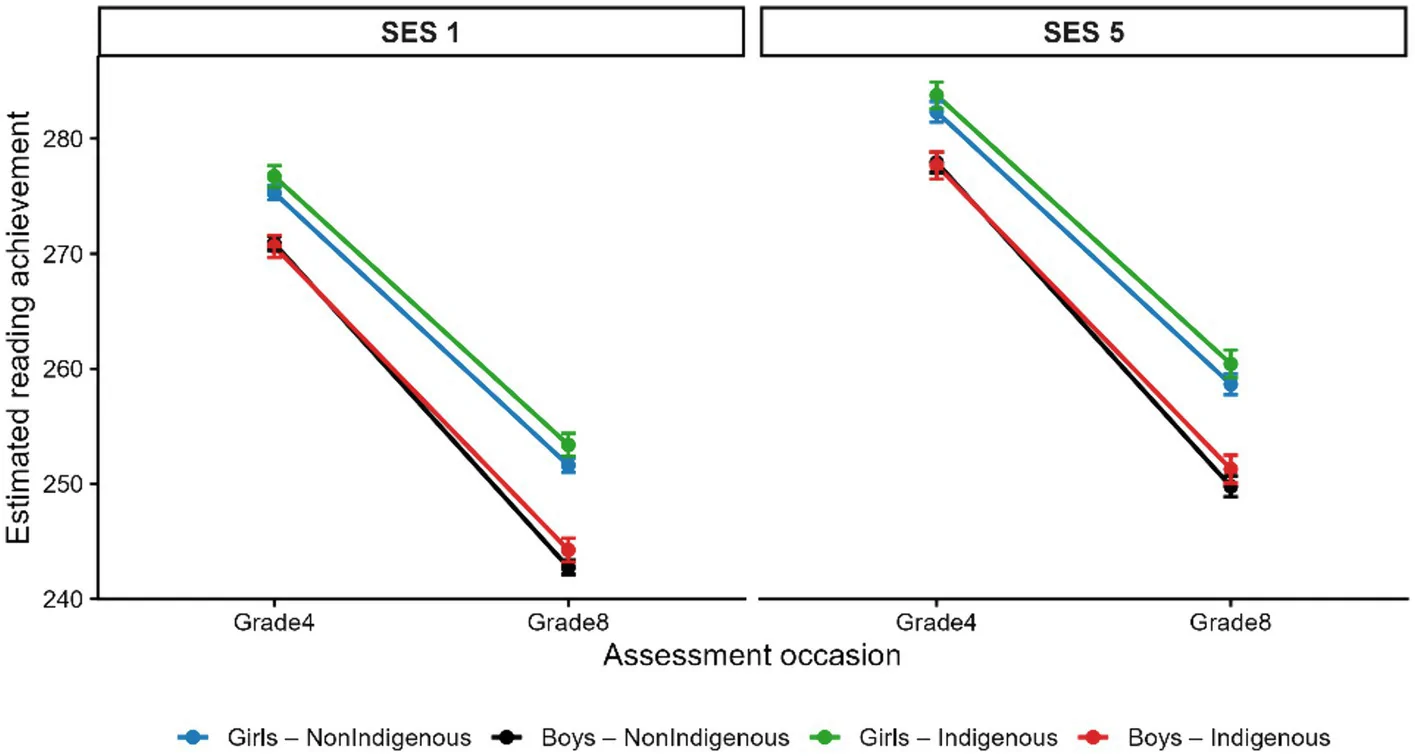
Estimated reading achievement change from Grade 4 (2015) to Grade 8 (2019) by socioeconomic status, student-reported sex, and Indigenous status. The figure presents estimated marginal means for students in the lowest (SES 1) and highest (SES 5) socioeconomic groups. Error bars represent 95% confidence intervals. Sex was reported by students in the Grade 4 student questionnaire, whereas Indigenous status and socioeconomic status were derived from the Grade 4 parent/guardian questionnaire. The figure should be interpreted as a complementary illustration because the Time × SES interaction was not statistically significant and model comparison favored the Time × Sex × Indigenous Status model.

Despite these level differences, the overall pattern across socioeconomic groups was similar. Model-based estimated reading scores were lower at the grade 8 assessment occasion than at the grade 4 assessment occasion in both SES1 and SES5, and the relative ordering of subgroups remained broadly stable: girls showed higher estimated scores than boys, and indigenous girls displayed the highest estimated scores within both socioeconomic groups. This pattern is consistent with the non-significant time × SES interaction and with the model comparison results, suggesting that socioeconomic status was associated mainly with differences in achievement levels rather than with differential longitudinal patterns of observed performance.

## 4 Discussion

### 4.1 Main findings

This study examined longitudinal change in reading achievement among Chilean students between Grade 4 and Grade 8 using a large national longitudinal dataset. Drawing on an ecological perspective, reading development was analyzed as a process associated with individual self-beliefs, family resources and expectations, school climate, and broader sociodemographic conditions. Overall, the findings support the view that reading achievement cannot be explained by a single domain, but is shaped by the joint contribution of psychological, family, school, and contextual factors (Bronfenbrenner, 1979; Chen et al., 2021; Claes et al., 2024; Mudrák et al., 2020; Yu et al., 2025). More specifically, the findings support the ecological proposition that longitudinal reading achievement is shaped by the continuous interaction between individual characteristics and multiple educational contexts rather than from isolated influences operating independently. This interpretation extends previous ecological accounts by examining these domains simultaneously within a national longitudinal framework.

The results showed lower observed reading scores at the Grade 8 assessment occasion than at the Grade 4 assessment occasion. This pattern should be interpreted cautiously because SIMCE Reading assessments are administered at different grade levels and reflect grade-specific curricular expectations. Therefore, the observed difference should not be interpreted as direct evidence of a causal decline in students’ underlying reading ability, but rather as lower observed performance in the second (Grade 8) SIMCE Reading assessment. Even with this caution, the finding underscores the importance of monitoring reading development during the transition from primary to lower secondary education, when students encounter more complex texts, greater academic demands, and changing motivational and school-related conditions (Larsen et al., 2022; Levlin et al., 2024; Preusler et al., 2022; Shen and Goodrich, 2024). This interpretation is compatible with developmental perspectives suggesting that reading trajectories reflect cumulative educational processes in which increasingly complex literacy demands interact with students’ motivational resources and contextual opportunities across schooling (Larsen et al., 2022; Shen and Goodrich, 2024; Torppa et al., 2024; Vasalampi et al., 2023).

Beyond the overall time pattern, the results highlight three central findings. First, academic self-concept showed a strong positive association with reading achievement, suggesting that students’ perceptions of their own academic competence are closely linked to their reading performance over time. Second, family resources and expectations, including parental education, cultural capital, parental educational expectations, and family reading attitudes, were positively associated with achievement, indicating that reading development remains strongly embedded in the home literacy and educational environment. Third, school climate and aggression-related factors showed a clear pattern: classroom order and engagement and school safety were positively associated with reading achievement, whereas school violence, peer aggression, perceived discrimination, school violence climate, and student–teacher aggression were negatively associated with performance. Collectively, these findings reinforce the view that longitudinal patterns of reading achievement are shaped by the interaction of supportive and adverse educational experiences accumulated across primary schooling, rather than through stable individual characteristics alone.

The moderation results further showed that longitudinal patterns differed according to the joint combination of Sex and Indigenous status, whereas socioeconomic status was mainly associated with achievement levels rather than with different patterns of change over time. This distinction is significant as it suggests that educational inequalities in reading achievement operate through multiple dimensions: some factors differentiate students’ overall performance levels, while others are associated with subgroup-specific patterns of change. These findings support an ecological and equity-oriented interpretation of reading development in which academic self-beliefs, family literacy conditions, school experiences, and structural inequalities jointly contribute to longitudinal reading achievement. Therefore, longitudinal patterns of reading achievement are better understood as the product of the combined influence of psychological, family, school, and structural conditions than as the result of isolated predictors. This interpretation is consistent with the ecological framework proposed in the Introduction and with recent evidence supporting integrated models of reading development (Chen et al., 2021; Claes et al., 2024; Mudrák et al., 2020; Wang and Hu, 2025; Xiao et al., 2024).

### 4.2 Academic self-concept and individual factors

At the individual level, academic self-concept showed a strong positive association with reading achievement. This finding reinforces the central role of students’ academic self-beliefs in reading development and is consistent with educational psychology research showing that perceived academic competence is closely related to achievement, motivation, engagement, and persistence in literacy-related tasks (Bandura, 1986; Basarkod et al., 2023; Geng et al., 2023; Wang, 2023). Students who perceive themselves as academically capable may be more likely to engage with demanding texts, persist when comprehension becomes difficult, and develop more favorable orientations toward school learning.

The current study findings are consistent with reciprocal models of academic development, according to which positive academic self-concept and reading achievement may mutually reinforce one another over time. Although the present study does not directly examine reciprocal effects, the observed longitudinal associations are compatible with previous evidence suggesting that achievement and academic self-beliefs influence one another across schooling (Cubillos and Troncoso, 2025; Symes et al., 2023; Vasalampi et al., 2023). Rather than representing a fixed personal attribute, academic self-concept appears to function as a developmental resource that is continuously shaped by students’ learning experiences and, in turn, influences subsequent engagement and academic performance (Chen et al., 2021; Geng et al., 2023; Wang, 2023).

The positive associations observed in the current study for academic engagement and self-efficacy, teacher regulation and normative guidance, and school safety perception further suggest that reading achievement is supported by both motivational and contextual conditions. These findings align with evidence indicating that reading self-beliefs may mediate or connect the influence of family and school contexts with achievement outcomes (Chen et al., 2021; Ma et al., 2022; Ma et al., 2023). Consequently, academic self-concept should not be interpreted only as an individual attribute, but as part of a broader ecology of learning in which students’ perceptions of competence are shaped by prior achievement experiences, instructional feedback, classroom regulation, teacher support, and the perceived safety of the school environment. This interpretation is also consistent with ecological theory, as students’ self-perceptions emerge through repeated interactions with family members, teachers, peers, and classroom environments rather than through isolated individual characteristics (Bronfenbrenner, 1979).

The present findings also suggest that academic self-concept may be one pathway through which family and school contexts become associated with reading achievement. Supportive instructional practices, positive family literacy environments, and well-regulated classrooms may strengthen students’ confidence in their academic abilities, thereby contributing to more favorable reading achievement over time.

The magnitude of the association between academic self-concept and reading achievement is particularly relevant for the Chilean context, where previous SIMCE-based evidence has shown that students’ self-efficacy and motivational dispositions are important factors associated with academic performance (Salvo-Garrido et al., 2023). The present findings extend this evidence longitudinally by showing that early academic self-beliefs measured in Grade 4 are associated with reading achievement across the transition to Grade 8. Therefore, strengthening students’ confidence, perceived competence, and engagement with reading may be especially important before and during the transition from primary to lower secondary education. This developmental transition may be particularly sensitive because students encounter increasingly demanding literacy tasks while simultaneously experiencing important academic, motivational, and social changes. Early positive academic self-beliefs may therefore provide an important psychological resource for maintaining engagement with increasingly complex reading demands across this period (Larsen et al., 2022; Preusler et al., 2022; Torppa et al., 2024; Vasalampi et al., 2023).

### 4.3 Family resources, expectations, and reading attitudes

Family-related factors were consistently associated with reading achievement. Parental education, cultural capital, parental educational expectations, and family reading attitudes showed positive associations with reading performance, indicating that reading development is strongly embedded in students’ home literacy and educational environments. These findings are consistent with ecological and family literacy perspectives, which emphasize that reading development is shaped by everyday family contexts in which children encounter books, language, educational expectations, and opportunities for literacy engagement (Bronfenbrenner, 1979; Claes et al., 2024; Colgate and Colgate, 2025; Dahl-Leonard et al., 2025; Yu et al., 2025). They also suggest that family influences extend beyond the provision of material resources, as home environments may shape students’ motivational orientations, academic self-concept, and long-term engagement with literacy activities.

The positive associations observed in the current study for parental education and cultural capital suggest that structural family resources remain important for reading achievement. Families with higher educational and cultural resources may be better positioned to provide access to books, literacy materials, language-rich interactions, and support for school learning (Gubbins and Otero, 2020; Mudrák et al., 2020; Tong et al., 2023). Consequently, longitudinal reading achievement is associated not only with students’ individual characteristics, but also with the material and cultural resources available in their family environments.

Parental educational expectations also emerged as an important family-related predictor. This finding is consistent with research indicating that high expectations may support students’ academic orientation, persistence, and beliefs about their capacity to succeed, particularly when they are accompanied by emotional support and learning opportunities (Gubbins and Otero, 2020; Mudrák et al., 2020; Psyridou et al., 2025; Salvo-Garrido et al., 2023). Family expectations may therefore contribute to reading development not only by encouraging achievement-oriented behaviors, but also by reinforcing students’ academic self-beliefs and long-term educational aspirations. This interpretation is also consistent with recent evidence suggesting that parental expectations communicate educational values and future aspirations that become incorporated into students’ academic identities and perceptions of competence over time (Colgate and Colgate, 2025; Ma et al., 2023).

Family reading attitudes were positively associated with reading achievement, highlighting the importance of the value attributed to reading at home. Families that encourage reading, talk about books, provide reading opportunities, or model positive attitudes toward literacy may help students develop stronger reading engagement and more favorable orientations toward reading tasks (Claes et al., 2024; Moon and Hofferth, 2016; Psyridou et al., 2025; Vasalampi et al., 2023). This result is particularly relevant as it suggests that family literacy environments matter beyond structural indicators such as parental education and socioeconomic status. These findings reinforce the distinction described in the introduction between structural family resources, such as parental education and cultural capital, and relational family processes, such as reading attitudes and educational expectations. Both dimensions appear to contribute to longitudinal reading achievement through complementary rather than competing mechanisms.

Overall, these findings indicate that family resources, expectations, and reading attitudes are key components of longitudinal reading achievement. In the Chilean context, where educational opportunities remain strongly stratified by socioeconomic background, parental education, cultural capital, and home literacy practices may represent mechanisms through which reading inequalities are reproduced over time. Moreover, the associations found in the current study also help identify possible points for intervention, including strengthening family reading practices, improving access to books, and supporting school–family collaboration around literacy development. Rather than operating independently, these family-related factors appear to interact with students’ psychological resources and school experiences, supporting the broader ecological interpretation proposed throughout this study.

### 4.4 School climate, violence, and peer aggression

School-related factors were also associated with longitudinal reading achievement. Classroom order and engagement and school safety perception were positively associated with reading performance, whereas school violence and peer aggression, perceived discrimination at school, school violence climate, and student–teacher aggression were negatively associated with achievement. These findings highlight the school microsystem as a central context for reading development, where safety, order, support, conflict, and discrimination may shape students’ opportunities to engage with learning. Consistent with Bronfenbrenner’s ecological perspective (Bronfenbrenner, 1979), school climate should be understood not merely as a contextual backdrop for learning, but as a developmental environment in which students construct academic confidence, engagement, and expectations of success through everyday interactions with teachers and peers.

The negative association between school violence and peer aggression and reading achievement suggests that exposure to aggressive peer interactions or violent school environments in Grade 4 may be linked to less favorable reading outcomes over time. The development of reading skills is supported by concentration, emotional security, persistence, and active engagement with texts; thus, violence, bullying, and peer aggression may weaken the cognitive and socioemotional conditions needed for sustained literacy development. This interpretation is consistent with previous Chilean evidence showing that school violence, bullying, and discrimination are negatively associated with standardized academic performance in Reading, Mathematics, and other domains (Bravo-Sanzana et al., 2022; Salvo-Garrido et al., 2020; Salvo-Garrido et al., 2023). Beyond reducing immediate learning opportunities, repeated exposure to hostile school environments may progressively weaken students’ motivation, academic self-beliefs, and willingness to engage with increasingly demanding literacy tasks, consistent with developmental perspectives emphasizing that adverse educational experiences may accumulate across schooling and shape reading trajectories over time (Larsen et al., 2022; Preusler et al., 2022; Wang and Hu, 2025).

Perceived discrimination at school also showed a negative association with reading achievement. This finding is key as discrimination may affect learning not only through direct academic barriers, but also through students’ sense of belonging, perceived fairness, emotional security, and trust in the school environment. Cross-national evidence has similarly shown that discriminatory school climates are associated with lower reading and mathematics performance, partly through reduced school belonging and a lower perceived value of learning (Baysu et al., 2023; Heppt et al., 2025). In the context of Indigenous and culturally minoritized students, this result underscores the importance of school environments that recognize students’ identities, languages, histories, and community-based forms of knowledge (Arias-Ortega and Villarroel-Cárdenas, 2024; Arias Ortega and Del Pino, 2025; Hayes et al., 2025; Torres Cuevas et al., 2024). More broadly, the finding reinforces the view that discriminatory school climates may undermine students’ academic belonging, perceived competence, and engagement with school learning (Baysu et al., 2023; Heppt et al., 2025; Xiao et al., 2024).

The negative associations observed for school violence climate and student–teacher aggression further indicate that hostile or conflictive school environments may hinder reading achievement. Conversely, classroom order and engagement were positively associated with reading performance, suggesting that organized and academically engaged classrooms may provide the structure needed for students to participate, concentrate, receive feedback, and sustain effort in reading tasks. These findings are consistent with research showing that teacher support, disciplinary climate, classroom regulation, and positive school relationships are associated with stronger academic engagement, self-concept, and reading outcomes (Chen et al., 2021; Ma et al., 2022; Tan et al., 2022; Wang and Hu, 2025).

Overall, these findings indicate that school climate and aggression-related factors are central components of longitudinal reading achievement. From an ecological perspective, schools are not only settings where reading instruction takes place; they are relational environments that can either support or constrain students’ academic confidence, emotional security, motivation, and engagement. Therefore, efforts to improve reading achievement should consider not only instructional practices, but also the reduction of school violence, the prevention of peer aggression, the recognition of discriminatory experiences, the strengthening of teacher–student relationships, and the promotion of orderly, supportive, and inclusive classrooms.

### 4.5 Sex, indigenous status, and socioeconomic inequalities in longitudinal Reading achievement change

The moderation results showed that longitudinal change in reading achievement differed according to the joint combination of student-reported sex and Indigenous status. Across both measurement occasions, girls showed higher estimated reading achievement than boys, regardless of Indigenous status. This pattern is consistent with international evidence showing that girls tend to outperform boys in reading and often report more favorable reading attitudes, stronger reading self-concept, greater reading enjoyment, and more frequent use of reading-related strategies (Acar-Erdol and Akin-Arikan, 2022; Huang and Chen, 2019; Reinhold et al., 2026; Taraszow et al., 2024). These differences in reading performance should not be interpreted as fixed ability differences, but as developmental patterns shaped by motivation, self-beliefs, socialization, engagement, and domain-specific academic identities (Acar-Erdol and Akin-Arikan, 2022; Reinhold et al., 2026; Steinmann and Strietholt, 2026).

The significant Time × Sex × Indigenous status interaction suggests that patterns of longitudinal change were not homogeneous across sociodemographic groups. Indigenous girls displayed the highest estimated reading scores at both time points, followed by non-Indigenous girls, whereas boys showed lower estimated achievement levels. Indigenous boys also showed slightly higher estimated scores than non-Indigenous boys in Grade 8. Importantly, these positive differences associated with Indigenous status should not be interpreted as indicating a general academic advantage of Indigenous students or as reflecting inherent group characteristics. The conditional coefficient for Indigenous status was small in magnitude, and its meaning depended on sex and assessment occasion within the retained interaction structure. One plausible interpretation is that the observed pattern may reflect heterogeneous combinations of family, cultural, linguistic, community, and school-related resources that are not fully captured by the variables available in the SIMCE database. Indigenous families and communities may provide culturally grounded literacy resources, including oral storytelling, community histories, bilingual practices, and other forms of knowledge that can support literacy development, while students may simultaneously experience structural and school-based inequalities. These patterns should be interpreted cautiously, because Indigenous status was modeled as a broad category and does not capture the diversity of Indigenous peoples, languages, territories, school contexts, or family literacy practices in Chile. Rather than suggesting a uniform “Indigenous effect,” the findings indicate that Indigenous family background is associated with reading achievement in context-dependent ways and in combination with sex and other ecological conditions.

From an equity perspective, understood as the fair distribution of educational opportunities and the recognition of students’ cultural and linguistic backgrounds, these results highlight the need to move beyond single-axis interpretations of educational inequality. Indigenous and culturally minoritized students may experience school environments in which their identities, languages, histories, and community-based forms of knowledge are insufficiently recognized or valued (Arias-Ortega and Villarroel-Cárdenas, 2024; Arias Ortega and Del Pino, 2025; Hayes et al., 2025; Torres Cuevas et al., 2024). At the same time, Indigenous families and communities may transmit rich literacy practices through oral storytelling, community histories, bilingual resources, and culturally grounded forms of knowledge (Hayes et al., 2025; Ramos Salazar et al., 2023).

Socioeconomic status was positively associated with reading achievement, indicating that students from more advantaged socioeconomic backgrounds showed higher estimated reading scores. However, model comparison did not support SES as an additional moderator of change over time, and the Time × SES interaction was not retained in the most parsimonious model. This suggests that SES was associated mainly with differences in achievement levels rather than with substantially different longitudinal patterns of change. Thus, socioeconomic inequalities were present across both assessment occasions, but they did not appear to redefine the overall direction of change between Grade 4 and Grade 8.

These findings suggest that reading achievement inequalities are configured through multiple, overlapping dimensions. SES continues to matter because it is linked to access to cultural capital, books, family literacy practices, parental education, and school resources (Claes et al., 2024; Colgate and Colgate, 2025; Ma et al., 2023; Yu et al., 2025). However, the longitudinal pattern was more clearly differentiated by the joint combination of Sex and Indigenous status, indicating that performance levels and subgroup-specific patterns of change should be interpreted as related but distinct dimensions of educational inequality.

### 4.6 Educational implications

Given the observational nature of the study, these implications should be interpreted as potential directions for educational practice and policy rather than as evidence of intervention effectiveness. The findings have several implications for educational practice, equity, social justice, and policy. First, reading interventions should address not only cognitive and instructional components, but also students’ academic self-beliefs. Although decoding, fluency, vocabulary, and comprehension strategies remain essential, the strong association between academic self-concept and reading achievement indicates that students’ perceptions of their own competence should also be considered in literacy development. Schools can support academic self-concept through constructive feedback, achievable challenges, opportunities for success, and classroom environments in which students experience themselves as capable readers (Bandura, 1986; Basarkod et al., 2023; Geng et al., 2023; Wang, 2023).

Second, the positive associations observed for parental education, cultural capital, parental educational expectations, and family reading attitudes suggest that reading development should be supported through school–family collaboration. Schools may promote family reading practices, facilitate access to books and literacy materials, and help families communicate high but realistic educational expectations. These actions are especially relevant in socially unequal contexts, where home literacy environments and cultural resources are unevenly distributed (Claes et al., 2024; Colgate and Colgate, 2025; Gubbins and Otero, 2020; Yu et al., 2025).

Third, the school climate findings indicate that literacy policies should be connected to efforts to promote safe, orderly, and inclusive learning environments. Reading development depends not only on instruction, but also on students’ sense of safety, belonging, emotional security, and willingness to participate in classroom activities. Therefore, interventions aimed at improving reading achievement should also seek to reduce school violence, prevent peer aggression, address discrimination, strengthen teacher–student relationships, and promote positive classroom organization (Baysu et al., 2023; Bravo-Sanzana et al., 2022; Ma et al., 2022; Salvo-Garrido et al., 2020; Su and Lee, 2023; Xiao et al., 2024).

Finally, subgroup differences by sex and Indigenous status point to the need for equity-oriented reading interventions. Boys may require targeted support to strengthen reading engagement, reading identities, and motivation, especially in contexts where reading is socially constructed as a less masculine domain (Acar-Erdol and Akin-Arikan, 2022; Reinhold et al., 2026; Steinmann and Strietholt, 2026). Interventions involving Indigenous students should avoid deficit-based interpretations and recognize culturally grounded literacy practices, oral traditions, bilingual resources, family knowledge, and community histories as valuable educational resources (Arias-Ortega and Villarroel-Cárdenas, 2024; Arias Ortega and Del Pino, 2025; Hayes et al., 2025; Torres Cuevas et al., 2024). Overall, improving reading achievement requires coordinated actions that integrate instruction, motivation, family engagement, positive school climate, cultural recognition, and equity-oriented resource allocation.

### 4.7 Strengths, limitations, and future research

This study has several strengths. First, it was based on a large national longitudinal sample of Chilean students, which provided substantial statistical power and made it possible to examine reading achievement between Grade 4 and Grade 8. By linking achievement records across two assessment cycles, the study moved beyond cross-sectional associations and analyzed longitudinal change in reading achievement across a key period of compulsory schooling. Second, the inclusion of individual, family, school-related, and sociodemographic predictors allowed reading achievement to be examined from an ecological perspective, rather than as the result of isolated factors. Third, the use of linear mixed-effects models accounted for the dependency between repeated observations within students and allowed the estimation of both overall changes in achievement and subgroup differences in longitudinal change.

Several limitations should also be considered. Because the study relied on observational secondary data, the associations identified in the models should not be interpreted as causal effects. In addition, all predictors were measured at baseline in Grade 4, which means that the analyses could not capture changes in academic self-concept, family reading attitudes, school climate, perceived discrimination, or other relevant factors between Grade 4 and Grade 8. Future studies should incorporate repeated measures of these predictors to examine reciprocal and dynamic relationships over time.

A further limitation is that reading achievement was measured at only two time points. Although this design allowed the study to examine change between primary and lower secondary education, it did not permit modeling nonlinear trajectories or identifying more complex patterns of stability or change across assessment occasions. Future longitudinal research should include additional measurement occasions to better characterize reading development across schooling. Moreover, Indigenous status was modeled as a broad category, which does not capture the diversity of Indigenous peoples, languages, territories, family literacy practices, or school contexts in Chile. Future studies should incorporate more culturally specific and context-sensitive indicators to better understand reading development among Indigenous and culturally minoritized students.

Finally, longitudinal attrition and complete-case inclusion may have introduced selection into the analytic sample. Sensitivity analyses indicated a moderate baseline reading achievement difference between students retained through Grade 8 and those lost to follow-up (*d* = 0.55), whereas differences in baseline sociodemographic characteristics were small. Among longitudinally retained students, differences between the final analytic sample and those excluded because of incomplete covariate data were small for baseline reading achievement (*d* = 0.19), sociodemographic characteristics, and continuous questionnaire-based predictors. Although these findings suggest comparatively modest selection on the observed covariates at the complete-case stage, longitudinal attrition was associated with baseline reading achievement, and missingness may also be related to unobserved factors such as student mobility or school changes. Future studies should further examine missing-data mechanisms and consider complementary approaches such as multiple imputation or sensitivity analyses.

## Data Availability

The data analyzed in this study is subject to the following licenses/restrictions: The datasets analyzed in this study are not publicly available due to confidentiality restrictions. Requests to access the datasets should be directed to the Chilean Education Quality Assurance Agency statistical information portal. Requests to access these datasets should be directed to https://informacionestadistica.agenciaeducacion.cl/#/bases.

## References

[ref1] Acar-Erdol T. Akin-Arikan Ç. (2022). Gender gap in reading achievement: the mediating role of metacognitive strategies and reading-related attitudes. Soc. Psychol. Educ. 25, 537–566. doi: 10.1007/s11218-022-09692-9, 30311153

[ref2] Arias Ortega K. Del Pino M. (2025). Survival experiences of Mapuche parents in multilevel schools in La Araucanía. J. Latinos Educ. 24, 662–675. doi: 10.1080/15348431.2024.2413522

[ref3] Arias-Ortega K. E. Villarroel-Cárdenas V. M. (2024). (in)justice in rural education within indigenous contexts in Chile. Magis 17, 1–21. doi: 10.11144/Javeriana.m17.ijse

[ref4] Bandura A. (1986). Social Foundations of Thought and action: A social Cognitive Theory. Englewood Cliffs, NJ: Prentice Hall.

[ref5] Basarkod G. Marsh H. W. Sahdra B. K. Parker P. D. Guo J. Dicke T. . (2023). The dimensionality of reading self-concept: examining its stability using local structural equation models. Assessment 30, 873–890. doi: 10.1177/10731911211069675, 35037486

[ref6] Baysu G. Agirdag O. De Leersnyder J. (2023). The association between perceived discriminatory climate in school and student performance in math and reading: a cross-national analysis using PISA 2018. J. Youth Adolesc. 52, 619–636. doi: 10.1007/s10964-022-01712-3, 36477568 PMC9884186

[ref7] Bravo Sanzana M. Salvo-Garrido S. Muñoz Poblete C. (2015). Profiles of Chilean students according to academic performance in mathematics: an exploratory study using classification trees and random forests. Stud. Educ. Eval. 44, 50–59. doi: 10.1016/j.stueduc.2015.01.002

[ref8] Bravo-Sanzana M. Bangdiwala S. I. Miranda R. (2022). School violence negative effect on student academic performance: a multilevel analysis. Int. J. Inj. Control Saf. Promot. 29, 29–41. doi: 10.1080/17457300.2021.1994615, 34719336

[ref9] Breiman L. (2001). Random forests. Mach. Learn. 45, 5–32. doi: 10.1023/A:1010933404324

[ref10] Bronfenbrenner U. (1979). The Ecology of human Development: Experiments by Nature and design. Cambridge, MA: Harvard University Press.

[ref11] Capin P. Roberts G. Clemens N. H. Vaughn S. (2022). When treatment adherence matters: interactions among treatment adherence, instructional quality, and student characteristics on reading outcomes. Read. Res. Q. 57, 753–774. doi: 10.1002/rrq.442, 35821988 PMC9272984

[ref12] Chen F. Sakyi A. Cui Y. (2021). Linking student, home, and school factors to reading achievement: the mediating role of reading self-efficacy. Educ. Psychol. 41, 1260–1279. doi: 10.1080/01443410.2021.1953445

[ref13] Claes R. Laga J. Denies K. Bleukx N. Dockx J. Van Keer H. . (2024). The interplay between students’ home literacy environment, reading attitudes and comprehension: a serial mediation analysis using PIRLS 2021-data. Large-Scale Assess. Educ. 12. doi: 10.1186/s40536-024-00233-8

[ref14] Colgate M. Colgate O. (2025). Insights and challenges for educational leaders supporting families in home reading practices. Educ. Sci. 15:296. doi: 10.3390/educsci15030296

[ref15] Cubillos M. Troncoso R. (2025). The evolving relationship between reading motivation and achievement: a longitudinal study. Educ. Sci. 15:1274. doi: 10.3390/educsci15101274

[ref16] Dahl-Leonard K. Hall C. Cho E. Capin P. Roberts G. J. Kehoe K. F. . (2025). Examining the effects of family-implemented literacy interventions for school-aged children: a meta-analysis. Educ. Psychol. Rev. 37:10. doi: 10.1007/s10648-025-09985-3

[ref17] Dimitropoulou P. Filippatou D. Gkoutzourela S. Griva A. Pachiti I. Michaelides M. (2025). The synergy of school climate, motivation, and academic emotions: a predictive model for learning strategies and reading comprehension. Behav. Sci. 15:503. doi: 10.3390/bs15040503, 40282123 PMC12024306

[ref18] Geng S. Lu Y. Shu H. (2023). Cross-cultural generalizability of expectancy-value theory in reading: a multilevel analysis across 80 societies. Curr. Psychol. 42, 18943–18958. doi: 10.1007/s12144-022-03014-0

[ref19] Genuer R. Poggi J.-M. Tuleau-Malot C. (2010). Variable selection using random forests. Pattern Recogn. Lett. 31, 2225–2236. doi: 10.1016/j.patrec.2010.03.014

[ref20] Gómez G. Rivas M. (2022). Reading achievement, resilience, and motivation in contexts of vulnerability: a study of perceived self-efficacy, intrinsic motivation, and family support in Chile. Read. Psychol. 43, 357–379. doi: 10.1080/02702711.2022.2106333, 37339054

[ref21] Gubbins V. Otero G. (2020). Parental involvement and low-SES children’s academic achievement in early elementary school: new evidence from Chile. Educ. Stud. 46, 548–569. doi: 10.1080/03055698.2019.1620691

[ref22] Hay I. Stevenson Y. M. (2024). The level of predictability of past reading attainment and self-perception and behavioural measures on children’s reading achievement. Aust. J. Educ. 68, 124–144. doi: 10.1177/00049441241253027

[ref23] Hayes C. Murnan R. Bequette S. (2025). Family, culture, and literacy: unlocking the power of home for early literacy achievement. Early Child. Educ. J. 54, 1971–1981. doi: 10.1007/s10643-025-01986-9

[ref24] Heppt B. Schwarzenthal M. Scharf J. (2025). Discriminatory climate and school adjustment in ethnically minoritized adolescents and majority adolescents: an investigation of the mediating role of teaching quality. J. Youth Adolesc. 54, 1732–1755. doi: 10.1007/s10964-025-02147-2, 39918709 PMC12245938

[ref25] Huang J. Chen G. (2019). From reading strategy instruction to student reading achievement: the mediating role of student motivational factors. Psychol. Sch. 56, 724–740. doi: 10.1002/pits.22217

[ref26] Lam C. B. Chung K. K. H. (2023). Maternal warmth moderates the longitudinal associations of family economic pressure with early reading and writing skills among Chinese children. Learn. Individ. Differ. 101:102246. doi: 10.1016/j.lindif.2022.102246

[ref27] Larsen S. A. Little C. W. Coventry W. L. (2022). The codevelopment of reading and attention from middle childhood to early adolescence: a multivariate latent growth curve study. Dev. Psychol. 58, 1017–1034. doi: 10.1037/dev0001344, 35311314

[ref28] Levlin M. Okonski L. Sullivan K. P. H. (2024). Educational attainment in Swedish (L1) and English (L2) for students with reading difficulties: a longitudinal case study from primary to the end of secondary school. Scand. J. Educ. Res. 68, 172–188. doi: 10.1080/00313831.2022.2123850

[ref29] Ma L. Xiao L. Hau K.-T. (2022). Teacher feedback, disciplinary climate, student self-concept, and reading achievement: a multilevel moderated mediation model. Learn. Instr. 79:101602. doi: 10.1016/j.learninstruc.2022.101602

[ref30] Ma L. Xiao L. Li Q. (2023). Mediation of self-concept and moderation of teacher support between SES and reading achievement: evidence from China and the United States. Br. J. Educ. Psychol. 93, 921–940. doi: 10.1111/bjep.12607, 37151177

[ref31] Maclean M. Lima F. O’Donnell M. (2024). Investigating positive reading trajectories among children who experienced out-of-home care. Child Youth Serv. Rev. 163:107728. doi: 10.1016/j.childyouth.2024.107728

[ref32] Moon U. J. Hofferth S. L. (2016). Parental involvement, child effort, and the development of immigrant boys’ and girls’ reading and mathematics skills: a latent difference score growth model. Learn. Individ. Differ. 47, 136–144. doi: 10.1016/j.lindif.2016.01.001, 26900304 PMC4755031

[ref33] Mudrák J. Zábrodská K. Takács L. (2020). Systemic approach to the development of reading literacy: family resources, school grades, and reading motivation in fourth-grade pupils. Front. Psychol. 11:37. doi: 10.3389/fpsyg.2020.00037, 32153445 PMC7045896

[ref34] Ohle-Peters A. McElvany N. Ullrich M. (2023). Students reading text-picture-material: the role of teacher competence and instructional quality. Int. J. Educ. Psychol. 12, 251–268. doi: 10.17583/ijep.11558

[ref35] Preusler S. Zitzmann S. Baumert J. Möller J. (2022). Development of German reading comprehension in two-way immersive primary schools. Learn. Instr. 79:101598. doi: 10.1016/j.learninstruc.2022.101598

[ref36] Psyridou M. Torppa M. Lervag A. Poikkeus A. M. Lerkkanen M. K. (2025). Promotive and protective effects of cognitive, motivational, and parental factors on reading fluency and reading comprehension in grade 9. Sci. Stud. Read. 29, 470–499. doi: 10.1080/10888438.2025.2544010

[ref37] Ramazan O. Dai S. Danielson R. W. Ardasheva Y. Hao T. Austin B. W. (2023). Students’ 2018 PISA reading self-concept: identifying predictors and examining model generalizability for emergent bilinguals. J. Sch. Psychol. 101:101254. doi: 10.1016/j.jsp.2023.101254, 37951665

[ref38] Ramos Salazar L. Diego-Medrano E. García N. Castillo Y. (2023). An examination of family cohesion and self-esteem as mediators of bilingualism and reading achievement among second-generation immigrant students. J. Latinos Educ. 22, 1660–1675. doi: 10.1080/15348431.2022.2030288

[ref39] Raudenbush S. W. Bryk A. S. (2002). Hierarchical linear Models: Applications and data Analysis Methods. 2nd Edn. Thousand Oaks, CA: Sage.

[ref40] Raufelder D. Steinberg O. Viljaranta J. Poikkeus A. M. Vasalampi K. (2024). A multigroup random-intercept cross-lagged panel model for Finnish secondary school students in frame of situated expectancy-value theory. Learn. Individ. Differ. 116:102555. doi: 10.1016/j.lindif.2024.102555

[ref41] Reinhold F. Diedrich J. Schiepe-Tiska A. León Gómez Yepes R. Hofer S. I. (2026). Gender effects in mathematics, science, and reading achievement: a secondary analysis of PISA data on the role of expectancies and values as well as cultural and societal norms. Learn. Individ. Differ. 127:102890. doi: 10.1016/j.lindif.2026.102890

[ref42] Ren R. Chen W. Zhao S. (2025). The indirect effects of school bullying on mathematics achievement: the mediating roles of teacher-student relationships, sense of belonging and differences between genders. BMC Public Health 25:113. doi: 10.1186/s12889-025-21307-4, 39789514 PMC11720600

[ref43] Salvo-Garrido S. Vargas H. Urra O. Gálvez-Nieto J. L. Miranda-Zapata E. (2020). Estudiantes resilientes en el área de matemática: Examinando los factores protectores y de riesgo en un país emergente. Rev. Iberoam. Diagn. Eval. Psicol. 55, 43–57. doi: 10.21865/ridep55.2.04

[ref44] Salvo-Garrido S. Zayas-Castro J. Polanco-Levicán K. Gálvez-Nieto J. L. (2023). Latent regression analysis considering student, teacher, and parent variables and their relationship with academic performance in primary school students in Chile. Behavioral Sciences 13:516. doi: 10.3390/bs13060516, 37366768 PMC10295613

[ref45] Shen Y. Goodrich J. M. (2024). Cross-linguistic influence of early Spanish reading on English reading trajectories among Spanish–English emergent bilinguals: a latent class growth analysis. J. Educ. Psychol. 116, 139–152. doi: 10.1037/edu0000824

[ref46] Shi Q. Erbeli F. Rice M. Butner J. E. (2023). The predictive role of early childhood dysregulation profile on the parallel growth trajectories of reading and math performance across elementary and middle school. Contemp. Educ. Psychol. 74:102200. doi: 10.1016/j.cedpsych.2023.102200

[ref47] Singer J. D. Willett J. B. (2003). Applied longitudinal data Analysis: Modeling change and event Occurrence. New York, NY: Oxford University Press.

[ref48] Steinmann I. Strietholt R. (2026). Reading more, achieving more? Long-term gender gaps in reading behavior and reading achievement. Stud. Educ. Eval. 88:101581. doi: 10.1016/j.stueduc.2026.101581

[ref49] Strobl C. Boulesteix A.-L. Zeileis A. Hothorn T. (2007). Bias in random forest variable importance measures: illustrations, sources and a solution. BMC Bioinformatics 8:25. doi: 10.1186/1471-2105-8-25, 17254353 PMC1796903

[ref50] Su M. Lee J. (2023). Eastern versus western gaps in whole child education: how school climate works for academic achievement and well-being across China, Korea, Finland, and the United States. Educ. Res. Policy Pract. 22, 117–138. doi: 10.1007/s10671-022-09322-3

[ref51] Symes W. Retelsdorf J. Lazarides R. (2023). Interindividual differences in the development of reading self-concept and intrinsic and importance value throughout secondary school. Contemp. Educ. Psychol. 75:102237. doi: 10.1016/j.cedpsych.2023.102237

[ref52] Tan Y. Fan Z. Wei X. Yang T. (2022). School belonging and reading literacy: a multilevel moderated mediation model. Front. Psychol. 13:816128. doi: 10.3389/fpsyg.2022.816128, 35185734 PMC8847447

[ref53] Tang X. Kikas E. Pakarinen E. Laursen B. Lerkkanen M. K. (2022). Longitudinal associations between third-grade teaching styles and sixth-grade reading skills: a 3-year follow-up study. J. Res. Read. 45, 157–169. doi: 10.1111/1467-9817.12385

[ref54] Taraszow T. Gentrup S. Heppt B. (2024). Egalitarian gender role attitudes give girls the edge: exploring the role of students’ gender role attitudes in reading and math. Soc. Psychol. Educ. 27, 3425–3452. doi: 10.1007/s11218-024-09913-3

[ref55] Tong C. K. Y. Ho J. C. S. Yang X. McBride C. Ng M. C. Y. Pan D. J. (2023). Transfer? Reading, writing, and parental factors in Hong Kong Chinese families across Chinese and English. Read. Writ. 36, 1997–2023. doi: 10.1007/s11145-022-10372-5

[ref56] Torppa M. Aro T. Eklund K. Parrila R. Eloranta A. K. Ahonen T. (2024). Adolescent reading and math skills and self-concept beliefs as predictors of age 20 emotional well-being. Read. Writ. 37, 2075–2099. doi: 10.1007/s11145-023-10461-z

[ref57] Torres Cuevas H. Quilaqueo Rapimán D. Baronnet B. Muñoz Troncoso G. (2024). Rethinking educational management of schools in a Mapuche context. Rev. Electron. Invest. Educ. 26, 1–15. doi: 10.24320/redie.2024.26.e01.5528

[ref58] Vasalampi K. Tolvanen A. Torppa M. Poikkeus A. M. Hankimaa H. Aunola K. (2023). PISA reading achievement, literacy motivation, and school burnout predicting adolescents’ educational track and educational attainment. Learn. Individ. Differ. 108:102377. doi: 10.1016/j.lindif.2023.102377

[ref59] Wang X. (2023). Chinese adolescents’ reading engagement profiles and their relations to self-concept and reading literacy. J. Res. Read. 46, 333–357. doi: 10.1111/1467-9817.12431

[ref60] Wang Y. Hu J. (2025). Relationships between reading engagement features and students’ digital reading performance: an international large-scale study. Learn. Instr. 99:102157. doi: 10.1016/j.learninstruc.2025.102157

[ref61] Xiao L. Ni A. Hau K. T. (2024). Moderation effects of teacher support and disciplinary climate on SES and reading relation in high and low globally competitive economies. Stud. Educ. Eval. 83:101389. doi: 10.1016/j.stueduc.2024.101389

[ref62] Yu L. Hou Y. Yu Y. Cheung H. Tong X. (2025). The longitudinal pathways linking SES to reading comprehension. J. Lit. Res. 57, 29–54. doi: 10.1177/1086296X251319464

