# Supplementary material for: Academic self-concept and ecological predictors of longitudinal change in reading achievement in Chile: evidence from a national study
Source: Front Psychol. 2026 Oct 12;17:1933540. doi: 10.3389/fpsyg.2026.1933540 (PMC13696404; doi:10.3389/fpsyg.2026.1933540)
Supplement: Supplementary file 1 [file Table_1.docx]

**Supplementary Table S1**

**Description and Reliability of Questionnaire-Based Indices Included in the Final Reading Achievement Model**

| **Variable/construct** | **Domain** | **Source/**  questionnaire | **Type scale** | **N° Items** | **Reliability (α,ω)** | **Interpretation** |
| --- | --- | --- | --- | --- | --- | --- |
| Academic Engagement and Self-Efficacy | Individual | Student | Likert | 9 | (.77,.77) | Higher scores indicate stronger academic engagement and perceived self-efficacy. |
| Academic Self-Concept | Individual | Student | Likert | 8 | (.80,.80) | Higher scores indicate more positive academic self-concept. |
| Teacher Regulation and Normative Guidance | Individual/school experience | Student | Likert | 5 | (.83,.83) | Higher scores indicate stronger perceived teacher regulation, guidance, and classroom norms. |
| School Safety Perception | Individual/school experience | Student | Likert | 2 | _ | Higher scores indicate greater perceived school safety. |
| School Violence and Peer Aggression | Individual/school experience | Student | Likert | 10 | (.93,.93) | Higher scores indicate greater perceived school violence and peer aggression. |
| Parental Education | Family | Parent/guardian | Ordinal | 2 | _ | Higher scores indicate higher parental educational attainment. |
| Cultural Capital (Books at Home) | Family | Parent/guardian | Ordinal | 1 | _ | Higher scores indicate a larger number of books at home. |
| Parental Educational Expectations | Family | Parent/guardian | Ordinal | 1 | _ | Higher scores indicate higher parental educational expectations. |
| Family Reading Attitudes | Family | Parent/guardian | Likert | 6 | (.84,.84) | Higher scores indicate more favorable family attitudes toward reading and home literacy-related practices. |
| Perceived Discrimination at School | Family/contextual | Parent/guardian | Likert | 10 | (.95,.95) | Higher scores indicate greater perceived discrimination or exclusion at school. |
| Classroom Order and Engagement | School | Teacher | Likert | 5 | (.85,.85) | Higher scores indicate greater classroom order and student engagement. |
| School Violence Climate | School | Teacher | Likert | 8 | (.91,.91) | Higher scores indicate greater perceived school violence climate. |
| Student–Teacher Aggression | School | Teacher | Likert | 4 | (.89,.89) | Higher scores indicate more frequent aggression between students and teachers. |

Note. All questionnaire-based indices were measured in Grade 4. The table reports the indices that were significant in the final reading achievement model. Multi-item indices were computed as the arithmetic mean of their constituent items, preserving the original response metric. For indices with three or more items, reliability was assessed using ordinal Cronbach’s alpha (α) and McDonald’s omega (ω), based on polychoric correlations. Reliability coefficients were not estimated for one- or two-item measures. Higher values indicate higher levels of the corresponding construct unless otherwise specified. Item codes and item wording are not reproduced due to data-use restrictions.
